# PD‐L1‐Binding Antigen Presenters: Redirecting Vaccine‐Induced Antibodies for Cancer Immunotherapy

**DOI:** 10.1002/advs.202519574

**Published:** 2026-02-11

**Authors:** Huixin Gao, Lijuan Lu, Xiaoxiao Xiong, Yi Li, Donghui Hu, Duo Zhang, Zhiwei Feng, Cong Liu, Nannan Liu, Xiaoli Li, Jizhou Tan, Ting Liu, Ling Peng, Lu Lu, Huiyi Feng, Yan Zhong, Guisen Tan, Zhicheng Zhang, Liqin Huang, Chao Su, Ying Xue, Shuo Song, Wenxia Fan, Wei Wang, Fan Zou

**Affiliations:** ^1^ Department of Clinical Laboratory Guangzhou Women and Children Medical Center Guangzhou Medical University Guangzhou Guangdong P.R. China; ^2^ Department of Medical Oncology the Third Affiliated Hospital of Sun Yat‐Sen University Guangzhou Guangdong P. R. China; ^3^ Faculty of Pharmaceutical Sciences Shenzhen University of Advanced Technology Shenzhen Guangdong P.R. China; ^4^ Department of Traditional Chinese Medicine The Third People's Hospital of Hubei Province Wuhan Hubei P.R. China; ^5^ The First Affiliated Hospital Sun Yat‐sen University Guangzhou P.R. China; ^6^ The Second Clinical Medical College Clinical Laboratory/State Key Laboratory of Traditional Chinese Medicine Syndrome Guangzhou University of Chinese Medicine Guangdong Provincial Hospital of Chinese Medicine Guangzhou P.R. China; ^7^ Beijing Luzhu Biotechnology Co. Ltd. Beijing P.R. China; ^8^ Department of Oncology Shenzhen Hospital of Southern Medical University Shenzhen Guangdong P.R. China; ^9^ Department of Pathology Shenzhen Hospital of Southern Medical University Shenzhen Guangdong P.R. China; ^10^ The First Affiliated Hospital of Guilin Medical University Guilin Guangxi P.R. China; ^11^ Zhongshan Institute for Drug Discovery Zhongshan Guangdong P.R. China; ^12^ Shenzhen Blood Center Shenzhen Guangdong P.R. China; ^13^ Jilin Province Jilin Hospital of Integrated Traditional Chinese and Western Medicine Jilin Jilin P.R. China; ^14^ Faculty of Life and Health Sciences Shenzhen University of Advanced Technology Shenzhen Guangdong P.R. China; ^15^ Department of Thoracic Surgery the Second Affiliated Hospital of Guangzhou Medical University Guangzhou Guangdong P.R. China

**Keywords:** antibody drug conjugates (ADC), antibody‐dependent cellular cytotoxicity (ADCC), PD‐L1 targeted immunotherapy, PD‐L1‐binding antigen presenter (PBAP), vaccine‐induced antibodies

## Abstract

The efficacy of immunotherapy in enhancing antitumor immunity in solid tumors remains limited, primarily due to the insufficient immunogenicity of tumor cells. In contrast, vaccination and natural viral infections can generate durable, high‐titer antiviral antibodies. A modular Programmed Death‐Ligand 1 (PD‐L1)‐binding antigen presenter (PBAP) has been engineered to tether varicella‐zoster virus (VZV) glycoprotein E (gE) to PD‐L1 expressed on tumor cell surfaces. This innovative construct leverages pre‐existing anti‐gE antibodies to trigger antibody‐dependent effector mechanisms. PBAP‐gE effectively bound to PD‐L1 positive tumor cells and, together with vaccine‐induced anti‐gE antibodies, potentiated NK cell‐mediated antibody‐dependent cellular cytotoxicity (ADCC) in vitro and induced significant tumor regression in murine models. The PBAP platform is modular and versatile. For example, a PBAP‐HER2 construct synergized with Herceptin and Kadcyla to eliminate human epidermal growth factor receptor 2 (HER2)‐negative, PD‐L1 positive cells. This work represents an innovative strategy for enhancing PD‐L1‐targeted therapies by leveraging pre‐existing antibodies induced by vaccination or natural viral infections, alongside commercially available antibody‐based therapies. Given the broad expression of PD‐L1 across various solid tumors and hematologic malignancies, our strategy holds promise as a potentially widely applicable platform for diverse PD‐L1‐positive patient populations.

## Graphical abstract

The PBAP‐gE complex anchors gE antigen to PD‐L1 on tumor cells. Vaccine‐induced anti‐gE antibodies simultaneously engage FcγRIIIa on NK cells and tumor‐bound PBAP‐gE, triggering NK cell activation and antibody‐dependent cellular cytotoxicity, thereby selectively eliminating PD‐L1–expressing tumor cells.

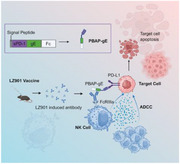

## 1 Introduction

Recent advances in high‐throughput technologies, particularly single‐cell sequencing, spatial transcriptomics, and multiplex immunohistochemistry, have profoundly expanded our understanding of the tumor microenvironment (TME), providing a more nuanced view of how tumors evade immune surveillance [1, 2, 3]. Despite the infiltration of various immune cell populations, such as cytotoxic T lymphocytes (CTLs) and natural killer (NK) cells, the ability of these cells to effectively recognize and eliminate tumor cells is often severely compromised. This dysfunction is primarily due to immune evasion mechanisms, such as antigen loss, impaired antigen presentation, and the establishment of an immunosuppressive TME [3, 4]. Notably, viral antigen‐specific bystander T cells within the TME often remain functionally competent but cannot effectively target tumor cells owing to the lack of tumor‐specific antigens [5, 6, 7, 8, 9, 10, 11, 12, 13, 14]. This highlights a critical gap in the immune response, where the presence of functional immune cells is insufficient to overcome tumor‐mediated immune escape.

To address these challenges, innovative therapeutic strategies—including the application of engineered oncolytic viruses (OVs) and bacteria—have emerged as promising platforms for personalized cancer vaccine development. These strategies aim to enhance immune recognition by “tagging” tumor cells with exogenous antigens. For example, OVs engineered to carry the model antigen ovalbumin (OVA), when combined with OVA‐specific OT‐1 T cells or OT‐1 peptide vaccines, have demonstrated synergistic antitumor efficacy in preclinical mouse models [15]. Beyond major histocompatibility complex (MHC)‐peptide presentation, OVs can also deliver large exogenous proteins to the surface of tumor cells. One representative example is an oncolytic Newcastle disease virus (NDV) engineered to express porcine α1,3‐galactosyltransferase (α1,3GT) on infected tumor cells, thereby inducing hyperacute rejection mediated by pre‐existing anti‐αGal antibodies and resulting in effective tumor elimination [16].

Similarly, a novel dual‐virus strategy has been developed in which OVs are engineered to drive the surface expression of truncated human epidermal growth factor receptor 2 (HER2) antigens on tumor cells. This approach enables the clinical application of HER2‐targeted antibody–drug conjugates (ADCs), such as ado‐trastuzumab emtansine (Kadcyla, also known as T‐DM1), even in HER2‐negative cancers, thereby overcoming therapeutic limitations imposed by the absence of endogenous HER2 expression [17]. Additionally, engineered bacteria delivering non‐tumor neoantigens have also shown promise in training the immune system to recognize and destroy cancer cells presenting identical antigens [18].

Importantly, accumulating evidence indicates that a substantial fraction of tumor‐infiltrating bystander T cells preferentially recognize virus‐associated antigens rather than tumor‐derived neoantigens [5, 6, 7, 8, 9, 10, 11, 12, 13, 14]. These bystander T cells play a critical role in shaping the TME and modulating antitumor immune responses. Studies have demonstrated that viral‐specific T cells, induced by Lymphocytic choriomeningitis virus (LCMV) infection or Severe acute respiratory syndrome coronavirus 2 (SARS‐CoV‐2) vaccination, can function as bystander T cells within the TME of tumor‐engrafted mice. Moreover, OVs engineered to express corresponding viral peptides can effectively “tag” tumor cells with viral antigens, thereby redirecting virus‐specific T cells to recognize and eliminate tumor cells in vivo [19]. Collectively, these findings support a tumor‐agnostic immunotherapeutic paradigm in which immunogenic, non‐tumor antigens are presented on tumor surfaces to recruit pre‐existing immune effectors and circumvent antigen loss–mediated immune escape. Consistent with this emerging paradigm, a recent study reported an intratumoral vaccination strategy in which tumor cells were chemically reprogrammed into antigen‐presenting like cells via Programmed death‐ligand 1 (PD‐L1) degradation‐coupled antigen presentation, thereby restoring effective antitumor immunity and further highlighting PD‐L1 as a strategic anchoring node for tumor‐directed immune reprogramming [20].

PD‐L1 serves as a central immune checkpoint regulator instrumental in tumor immune evasion. Through its interaction with the programmed death‐1 (PD‐1) receptor expressed on CTLs and NK cells, PD‐L1 delivers inhibitory signals that attenuate immune effector functions, thereby facilitating tumor escape from immune surveillance. Notably, PD‐L1 overexpression is a pervasive feature across a diverse array of malignancies, including solid tumors—such as non–small cell lung cancer (NSCLC), melanoma, and triple‐negative breast cancer—as well as hematologic cancers, notably Hodgkin lymphoma. This widespread upregulation is widely recognized as a pivotal mechanism by which human tumors actively subvert antitumor immunity. Moreover, PD‐L1 is also abundantly expressed not only on tumor cells but also on immunosuppressive cell populations, such as myeloid‐derived suppressor cells (MDSCs) and regulatory T cells (Tregs), as well as antigen‐presenting cells, including dendritic cells and macrophages, collectively positioning PD‐L1 as a strategic and multifaceted target for cancer immunotherapy [21, 22].

Therapeutic agents targeting PD‐L1, including monoclonal antibodies and ADCs, not only function through immune checkpoint blockade, which relieves inhibition of T cells, but also directly kill tumor cells via ADCC and the targeted delivery of cytotoxic agents by ADCs [23, 24, 25, 26, 27]. Nevertheless, the objective response rates achieved by PD‐L1 blockade monotherapy in solid tumors are still relatively low, highlighting the urgent need for more effective therapeutic strategies [28, 29]. Thus, the development of next‐generation PD‐L1‐targeted modalities, such as ADCs and bispecific antibodies, has gained increasing attention [23, 30, 31]. Several PD‐L1‐targeting ADCs, including SGN‐PDL1V (PF‐08046054), HLX43 (Henlius), and DB‐1419 (DualityBio), are currently undergoing clinical evaluation, offering a promising avenue to augment the current treatment armamentarium [32, 33, 34, 35].

Building upon the emerging concept of “tagging tumor cells with viral antigen peptides” to repurpose bystander T cells for tumor eradication [19], we sought to further enhance tumor‐targeted immune responses by exploiting pre‐existing humoral immunity. This strategy leverages the long‐term persistence of antibodies elicited by common vaccinations—such as measles, mumps, rubella, varicella‐zoster virus (VZV), *Clostridium tetani*, and *Corynebacterium diphtheriae*—as well as ubiquitously infecting viruses such as Epstein–Barr virus (EBV). Notably, vaccine‐ or infection‐induced antibodies against these pathogens remain detectable for decades, even in cancer patients [36, 37, 38, 39, 40, 41, 42].

**SCHEME 1:**
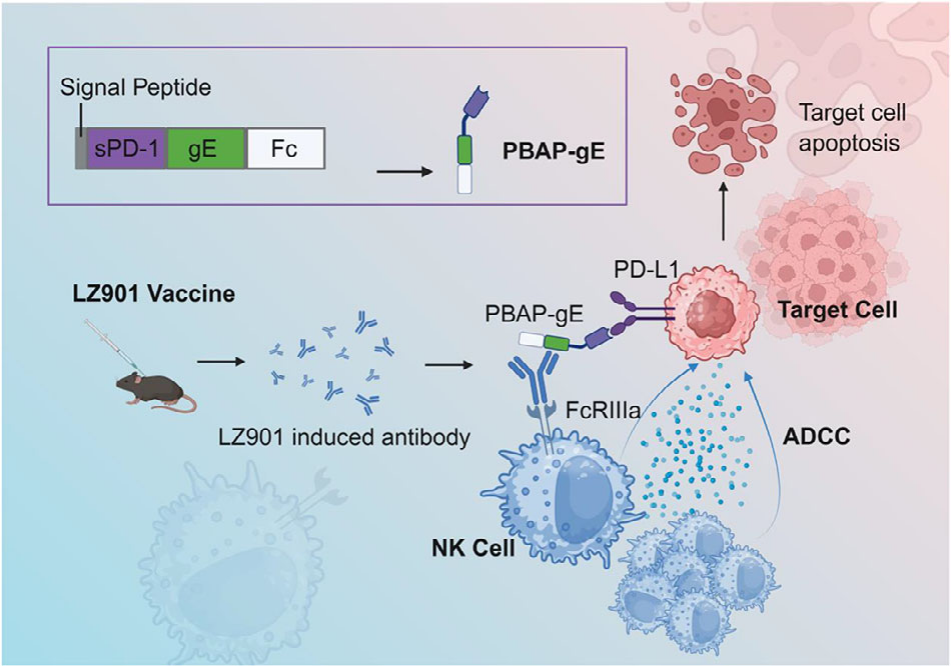
Mechanism of PBAP‐gE Complex Combined with LZ901 Vaccine in Enhancing NK Cell‐Mediated Antitumor Efficacy. The PBAP‐gE complex specifically binds to PD‐L1 on PD‐L1‐positive tumor cells via its sPD‐L1 domain, thereby labeling these cells with the gE antigen. Subsequently, the LZ901 vaccine activates the immune system to produce gE—specific antibodies (anti‐gE antibodies), which exert their effects through two distinct pathways. First, these antibodies directly bind to the FcγRIIIa receptors on NK cells, providing activation signals. Second, they specifically bind to the PBAP‐gE complex already present on tumor cells. Together, these dual actions trigger NK cell‐mediated ADCC, significantly augmenting NK cells’ ability to target and destroy PD‐L1‐positive tumor cells. Created with BioRender.com.

In prior experiments, we employed the recombinant zoster protein vaccine LZ901 (Beijing Luzhu Biotechnology Co., Ltd.), which has been validated to exhibit robust immunogenicity in both mice and humans [43, 44]. In tumor‐bearing C57BL/6 mice immunized with LZ901, we observed enrichment of B cells within tumor tissues. Notably, tumor‐infiltrating B cells produced significantly higher levels of anti‐VZV glycoprotein E (gE) IgG antibodies than B cells isolated from the spleen (Figure S1A,B). Drawing from these findings, we developed a modular platform, termed PD‐L1‐binding antigen presenter (PBAP), that anchors viral antigen to tumor cells via binding to PD‐L1. As a proof of concept, we engineered PBAP‐gE by fusing the extracellular domain of PD‐1 (soluble PD‐1, sPD‐1) to the VZV gE, together with an Fc domain to enhance stability. This platform leverages two key principles: (i) PD‐L1 is broadly expressed across diverse tumor types, and (ii) VZV vaccination elicits durable anti‐gE antibody responses in most adults. Collectively, this strategy enables antibodies generated through vaccination or natural infection to selectively recognize tumor cells and redirect pre‐existing immune responses toward tumor elimination (Scheme 1).

## 2 Results

### 2.1 PBAP‐gE Combined With Vaccine‐Induced Endogenous Antibodies Exhibits Antitumor Activity in Vitro

**FIGURE 1:**
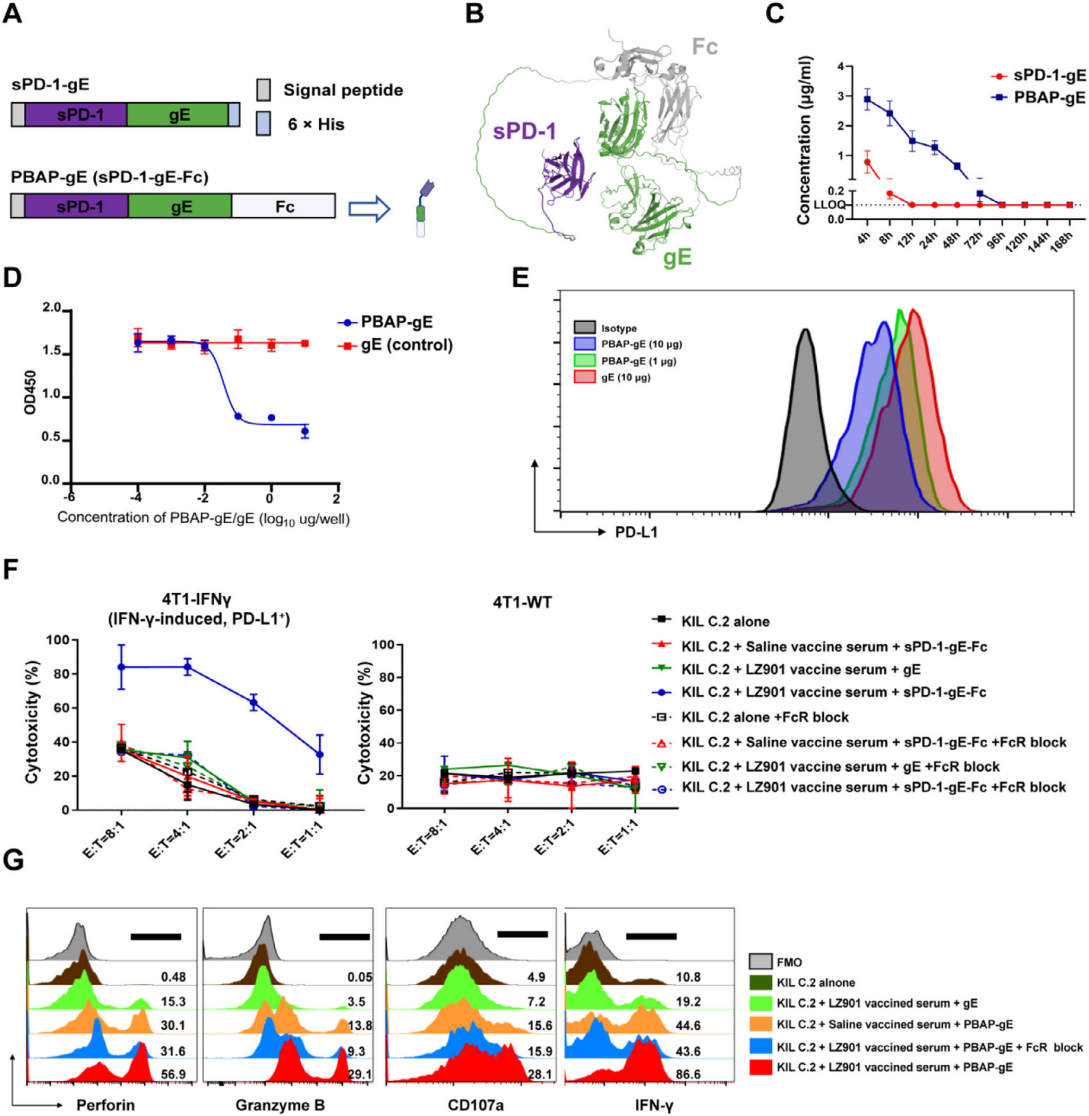
Serum from Herpes Zoster Vaccine (LZ901)‐immunized Mice Enhances PBAP‐Mediated ADCC Against PD‐L1^+^ Tumor Cells In Vitro. (A) Schematic diagram of the sPD‐1‐gE and PBAP‐gE fusion protein. The sPD‐1‐gE construct consists of sPD‐1 fused to gE. The PBAP‐gE construct comprises sPD‐1‐gE fused with an Fc domain (sPD‐1‐gE‐Fc). (B) Structural modeling of PBAP‐gE with AlphaFold 3. (C) Pharmacokinetic profiles of sPD‐1‐gE and PBAP‐gE following intravenous injection into C57BL/6J mice (n=3 mice/group, 100 µg/mice). Data are presented as the mean ± SD (n = 3). (D) The binding inhibition of PBAP‐gE on PD‐L1/PD‐1 interaction was assessed by ELISA. The absorbance was measured at 450 nm to determine the blocking effect. (E) The fluorescence intensity of the antibody‐cell binding was analyzed using a flow cytometry to assess the blocking effect on the PD‐1/PD‐L1 pathway. Data are presented as the mean ± SD (n = 3). (F) In vitro cytotoxicity assay. KIL C.2 cells were co‐incubated with PBAP‐gE and serum from LZ901‐immunized mice, against 4T1‐IFNγ (IFN‐γ‐induced, PD‐L1^+^) tumor cells and 4T1‐WT cells. KIL C.2 cells, KIL C.2 cells co‐incubated with gE and serum from LZ901‐immunized mice, KIL C.2 cells co‐incubated with PBAP‐gE and serum from saline vaccine‐immunized mice and all groups treated with anti‐FcγRIII blocking antibody were used as controls. Data are presented as mean ± SD of 3 independent experiments, each performed in triplicate. (G) Flow cytometry analysis of perforin, granzyme B, IFN‐γ and CD107a in KIL C.2 cells. Representative of 3 independent experiments.

The PBAP‐gE fusion protein was constructed by linking the extracellular domain of murine PD‐1 (sPD‐1) to gE via a flexible linker. An Fc domain was appended to the C‐terminus to extend the circulatory half‐life and enhance protein stability and bioactivity (Figure 1A; Figure S2A,B). Structural modeling of PBAP‐gE using AlphaFold 3 revealed no significant steric hindrance between the sPD‐1 and the gE domains, indicating that the fusion protein adopts a structurally compatible and well‐resolved configuration (Figure 1B). Pharmacokinetic analysis demonstrated that PBAP‐gE remained detectable in circulation for over 72 h in vivo, whereas the non‐Fc‐fused sPD‐1‐gE protein exhibited a markedly reduced half‐life of approximately 8 h (Figure 1C). To validate that PBAP‐gE competitively interferes with the binding of PD‐L1 to PD‐L1 antibodies in vitro, recombinant PD‐L1 protein and 4T1 cells engineered to stably overexpress murine PD‐L1 (designated 4T1‐PD‐L1‐OE) via lentiviral transduction and subsequent enrichment were employed. Enzyme‐linked immunosorbent assay (ELISA) and flow cytometric analyses confirmed that PBAP‐gE effectively interfered with the interaction between PD‐L1 and PD‐L1‐specific antibodies, both in solution and on the tumor cell surface (Figure 1D,E).

To generate gE‐specific hyperimmune serum for subsequent in vitro assays, C57BL/6 mice were immunized with LZ901 at a dose of 5 µg per mouse on days 0 and 21. On day 28, serum samples were collected via retro‐orbital bleeding for subsequent assays. The in vitro antitumor activity of the PBAP‐gE construct in combination with vaccine‐induced antibodies was initially evaluated using a lactate dehydrogenase (LDH) release assay. KIL C.2 cells, a murine NK cell line, were incubated with serum from LZ901‐immunized mice and subsequently co‐cultured with 4T1‐PD‐L1‐OE target cells pre‐incubated with PBAP‐gE. Under these conditions, KIL C.2 cells exhibited significantly enhanced cytotoxicity against tumor cells (Figure S3A). In contrast, negligible cytotoxicity was observed in control conditions, including NK cells alone, NK cells treated with gE protein plus immune serum, or NK cells treated with PBAP‐gE in the presence of serum from saline‐immunized mice.

To further confirm PD‐L1 dependency, a PD‐L1–knockout 4T1 cell line (4T1‐PD‐L1‐KO) was generated using CRISPR–Cas9. In this setting, PBAP‐gE in combination with immune serum failed to enhance NK cell–mediated cytotoxicity, demonstrating that tumor cell PD‐L1 expression is required for PBAP‐mediated immune engagement (Figure S3B), highlighting the essential role of PD‐L1 expression on tumor cells for PBAP‐mediated immune engagement.

To better recapitulate physiological conditions, we next examined wild‐type 4T1 cells, which exhibit minimal basal PD‐L1 expression, and the same cells treated with interferon‐γ to induce endogenous PD‐L1 expression (4T1‐IFNγ) (Figure S3C). PBAP‐gE combined with LZ901‐induced serum failed to induce lysis of 4T1‐WT cells but elicited potent cytotoxicity against 4T1‐IFNγ cells, comparable to that observed in 4T1‐PD‐L1‐OE cells (Figure 1F). Importantly, blockade of Fcγ receptors using an anti‐FcγRIII blocking antibody completely abrogated cytotoxicity, indicating that tumor cell killing was mediated exclusively by antibody‐dependent, NK cell‐driven ADCC rather than direct cytotoxicity.

Consistent with the mechanism mediated by ADCC, flow cytometric analysis of KIL C.2 cells revealed significantly increased expression of perforin, granzyme B, interferon‐γ, and CD107a following PBAP‐gE and LZ901‐immuned serum treatment, indicative of robust NK cell activation. This polyfunctional activation profile was entirely abolished by FcγRIII blockade (Figure 1G; Figure S4), confirming that PBAP‐gE licenses NK cell effector functions strictly through Fc receptor‐dependent ADCC.

### 2.2 PBAP‐gE Delivered via CAR‐T Cells and Intratumoral Administration Exhibits Potent Antitumor Activity in Vivo

Building on our previous work demonstrating that chimeric antigen receptor T (CAR‐T) cells can be engineered through multiple strategies—including inhibitory receptor knockout, functional protein overexpression, and combinatorial therapeutic regimens—to eradicate virus‐infected cells and solid tumors in both preclinical models and clinical settings, we explored the feasibility of using CAR‐T cells as a targeted delivery vehicle for PBAP‐gE [45, 46, 47, 48, 49, 50]. In contrast to oncolytic virus‐based platforms, CAR‐T cells enable spatially and temporally restricted payload release, as PBAP‐gE expression can be tightly coupled to CAR signaling. This strategy minimizes off‐target exposure and reduces the likelihood of circulating PBAP‐gE being neutralized by pre‐existing vaccine‐induced antibodies. Moreover, as a “living drug,” CAR‐T cells possess the capacity for long‐term persistence and immunological surveillance.

**FIGURE 2:**
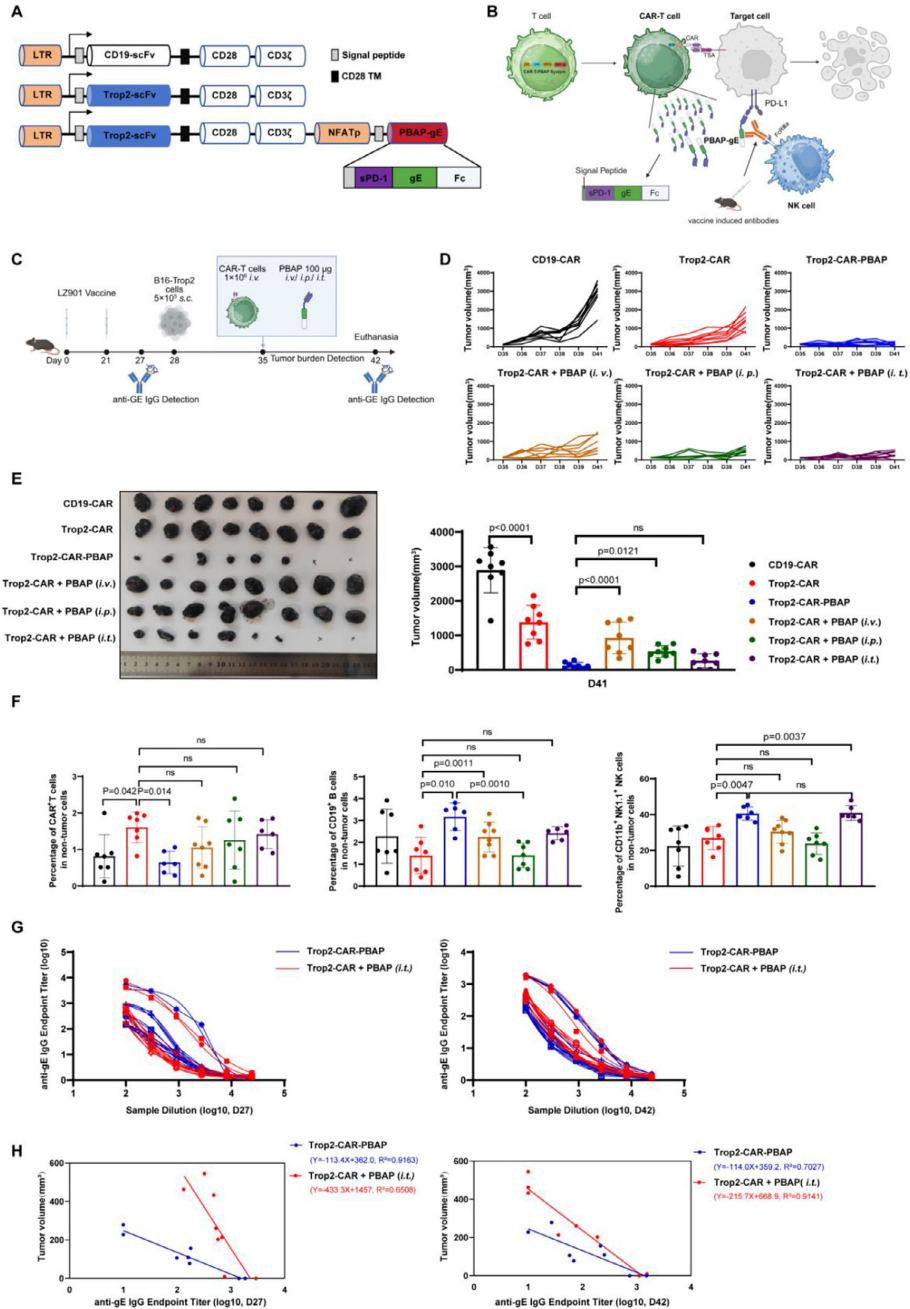
Trop2‐CAR‐T Cells Coexpressing PBAP‐gE or Combined with Intratumoral Injection of PBAP‐gE Induces Tumor Regression in LZ901‐Vaccinated Mice. (A) Schematic of CAR constructs. The control CAR fuses scFv VH/VL domains to the CD28 transmembrane (TM) and CD3ζ signaling domains. The experimental CAR adds a C‐terminal PBAP‐gE module under NFAT promoter control for tumor‐inducible expression. (B) Schematic of the CAR‐T‐PBAP system. Upon tumor antigen recognition by the CAR ectodomain, NFAT‐driven expression and secretion of PBAP‐gE recruits vaccine‐elicited or virus‐induced antibodies to FcγRIIIa^+^ NK cells, bridging them to PD‐L1^+^ tumor cells to trigger ADCC‐mediated lysis. Created with BioRender.com. (C) Experimental Design. C57BL/6J mice (n = 8 mice/group) received LZ901 vaccine (5 µg/dose) on days 0 and 21; Serum was collected on day 27 for anti‐gE IgG detection. B16‐Trop2 cells (5 × 10^5^) were subcutaneously implanted On day 28. When tumors reached ∼100 mm^3^ (day 35), mice were treated with a single infusion of CAR‐T cells (± PBAP‐gE) via intravenous (*i.v*.), intraperitoneal (*i.p*.), or intratumoral (*i.t*.) injection. Endpoint serum samples were collected on day 42 for anti‐gE IgG analysis. Created with BioRender.com. (D) Tumor volumes were measured every 1‐2 days from day 35 to day 41 in all groups, and all mice were euthanized on day 41. Tumor growth curves were plotted for the five experimental groups: CD19‐CAR, Trop2‐CAR, Trop2‐CAR‐PBAP, Trop2‐CAR + PBAP (*i*.*v*.), Trop2‐CAR + PBAP (*i*.*p*.), Trop2‐CAR + PBAP (*i*.*t*.). (E) Representative tumor images are shown in the left panel, and tumor volumes at the experimental endpoint are presented in the right panel. The PBAP‐coexpressing CAR‐T cell group exhibited tumor regression that was not statistically different from that in the intratumoral injection group. Data are presented as the mean ± SD, (n = 6–8). Statistical significance was determined using one‐way ANOVA. ns indicates not significant (p > 0.05). (F) Flow cytometric analysis of tumor‐infiltrating immune cells revealed that both the Trop2‐CAR‐PBAP and Trop2‐CAR + PBAP (*i*.*t*.) groups exhibited significantly elevated frequencies of B cells and NK cells. Data are presented as the mean ± SD, (n = 8). Statistical significance was determined using one‐way ANOVA. ns indicates not significant (p > 0.05). (G) ELISA was used to analyze the binding affinity of serum (collected on days 27 and 42) from LZ901‐vaccinated mice to gE in both the Trop2‐CAR‐PBAP and Trop2‐CAR + PBAP treatment groups. Data are presented as the mean ± SD (n = 8). (H) Correlation analysis was performed to examine the relationship between tumor volume and gE‐specific IgG antibody levels (endpoint titer) at days 27 and 42 in the Trop2‐CAR‐PBAP and Trop2‐CAR + PBAP‐gE (*i*.*t*.) treatment groups.

To achieve activation‐dependent PBAP‐gE expression, we constructed a multicistronic lentiviral system in which the CAR cassette—encoding either an anti‐Trop2 single‐chain variable fragment (scFv) or an anti‐CD19 scFv (serving as a control) —was constitutively expressed, whereas PBAP‐gE expression was placed under the control of an NFAT‐responsive promoter, thereby restricting PBAP‐gE secretion to activated CAR‐T cells (Figure 2A). Although CAR‐T therapies have previously been combined with chemotherapy, radiotherapy, and immune checkpoint inhibitors, their functional integration with NK cells has remained largely unexplored. Here, we conceptualized a dual‐effector strategy in which CAR‐T cells deliver PBAP‐gE to “tag” tumor cells, enabling vaccine‐induced antibodies to recruit NK cells for antibody‐dependent cytotoxicity (Figure 2B).

The in vitro properties of Trop2‐CAR‐T cells with or without PBAP‐gE expression (Trop2‐CAR‐PBAP and Trop2‐CAR, respectively) were first characterized. Trop2‐CAR‐PBAP cells exhibited a modest, non‐significant reduction in lentiviral transduction efficiency and CAR surface expression compared with Trop2‐CAR cells, likely reflecting the increased vector size introduced by the PBAP‐gE cassette (Figure S5A). Using B16 melanoma cells engineered to stably express Trop2 (B16‐Trop2), both Trop2‐CAR and Trop2‐CAR‐PBAP cells displayed comparable cytotoxic activity, indicating that PBAP‐gE incorporation did not compromise CAR‐mediated tumor cell lysis (Figure S5B). Furthermore, PBAP‐gE secretion was minimal when Trop2‐CAR‐PBAP cells were co‐cultured with Trop2‐negative B16‐F10 cells, whereas robust PBAP‐gE release was observed upon engagement with B16‐Trop2 cells, consistent with antigen‐dependent CAR activation (Figure S5C).

The therapeutic efficacy of this dual‐targeting strategy was next evaluated in syngeneic tumor models. B16‐Trop2 cells were subcutaneously implanted into C57BL/6 mice, which had been previously immunized with LZ901. Once tumors reached approximately 100 mm^3^, mice received intravenous infusion of CAR‐T cells and PBAP‐gE was administered either intravenously (*i.v*.), intraperitoneally (*i.p*.), or intratumorally (*i.t*.), depending on the experimental group (Figure 2C). Both Trop2‐CAR‐PBAP cells and intratumoral PBAP‐gE administration in combination with Trop2‐CAR cells resulted in marked tumor growth inhibition and regression. In contrast, systemic PBAP‐gE delivery via *i.v*. or *i.p*. routes produced only modest therapeutic effects (Figure 2D,E).

Analysis of tumor‐infiltrating immune populations revealed comparable CAR‐T cell infiltration across treatment groups, although absolute CAR‐T cell numbers were reduced in the Trop2‐CAR‐PBAP group relative to Trop2‐CAR alone. This reduction may reflect, at least in part, the expression of PD‐L1 on activated CAR‐T cells, rendering them susceptible to PBAP‐gE mediated antibody‐dependent clearance. Notably, both the Trop2‐CAR‐PBAP and Trop2‐CAR plus PBAP‐gE (*i.t*.) groups exhibited significantly increased infiltration of B cells and NK cells (Figure 2F, Figure S6). Importantly, tumor burden inversely correlated with serum gE‐specific IgG titers at both early and late time points, implicating antibody‐dependent NK cell‐mediated cytotoxicity as a dominant effector mechanism (Figure 2G,H; Figure S7).

Collectively, these results demonstrate that CAR‐T–mediated or intratumoral delivery of PBAP‐gE effectively integrates adaptive and innate immune mechanisms, resulting in superior antitumor activity in vivo.

### 2.3 Vaccine‐Induced Antibodies, Rather Than Virus‐Specific T Cells, Are the Primary Effectors in PBAP‐gE Mediated Tumor Suppression

The recombinant zoster vaccine LZ901 has been shown to elicit both robust humoral and cellular immune responses, including high titers of gE‐specific antibodies as well as strong CD4^+^ and CD8^+^ T‐cell responses, which together contribute to protection against VZV infection [43, 44]. Given that PBAP‐gE can potentially be internalized and processed by antigen‐presenting cells for presentation via MHC class I pathways, we sought to delineate the relative contributions of vaccine‐induced antibodies versus gE‐specific CD8^+^ T cells to PBAP‐gE–mediated antitumor efficacy.

**FIGURE 3:**
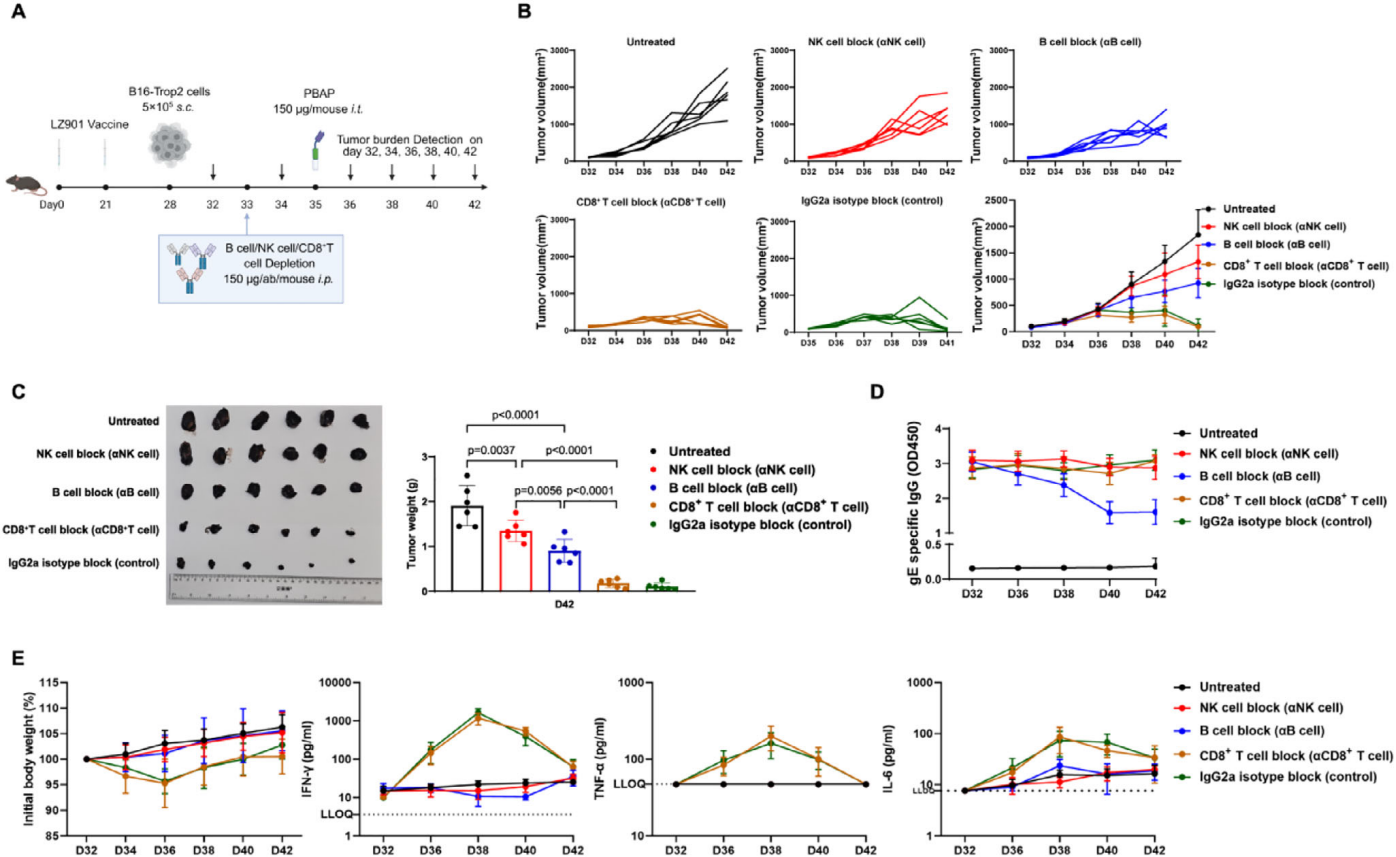
PBAP‐gE Elicits a Robust Antitumor Immune Response, Predominantly Mediated by NK Cells through gE‐Specific Antibody Production by B Cells. (A) Experimental design to assess the contributions of antibody versus CD8^+^ T cells in PBAP‐gE‐mediated tumor suppression. On day 0, C57BL/6J mice (n=5 mice/group) were initially vaccinated with the LZ901 vaccine (5 µg/dose), followed by a booster immunization on day 21. On day 28, a subcutaneous inoculation of 5×10^5^ B16‐Trop2 tumor cells was performed to establish the tumor model. On day 33, immune cell depletion was conducted via intraperitoneal injection of antibodies targeting NK cells (anti‐NK1.1), B cells (anti‐CD19), and CD8^+^ T cells (anti‐CD8), respectively. On day 34, each mouse received an intratumoral injection of 150 µg PBAP‐gE. Subsequently, tumor growth was monitored via bioluminescence imaging on days 36, 38, 40, and 42. (B) Tumor volumes were measured every 2 days from day 32 to day 42 in all groups, and all mice were euthanized on day 42. Tumor growth curves were plotted for the five experimental groups: untreated, NK cell block (αNK cell), B cell block (αB cell), CD8^+^ T cell block (αCD8^+^ T cell), IgG2a isotype block (control). Corresponding changes of tumor volum levels are shown on the right‐down panel. (C) Representative images of excised tumors are displayed in the left panel, and tumor weights (measured at the experimental endpoint on day 42) are presented in the right panel. Mice in the B cell depletion (αB cell) or NK cell depletion (αNK cell) groups exhibited significantly greater tumor weights compared to those in the CD8^+^ T cell depletion (αCD8^+^ T cell) group. In contrast, tumor weights in the CD8^+^ T cell depletion group were comparable to those in the IgG2a isotype control group. Data are presented as the mean ± SD (n = 6). Statistical significance was determined using one‐way ANOVA. ns indicates not significant (p > 0.05). (D) The gE‐specific IgG antibody levels (OD450) were measured every 2‐4 days from day 32 to day 42 across five experimental groups: Untreated, NK cell depletion (αNK cell), B cell depletion (αB cell), CD8^+^ T cell depletion (αCD8^+^ T cell), and IgG2a isotype control. Data are presented as the mean ± SD (n = 6). (E) Kinetics of initial body weight changes and serum levels of interferon‐gamma (IFN‐γ), tumor necrosis factor‐α (TNF‐α), and interleukin‐6 (IL‐6) across five experimental cohorts: Untreated, NK cell depletion (αNK cell), B cell depletion (αB cell), CD8^+^ T cell depletion (αCD8^+^ T cell), and IgG2a isotype control. Data are presented as the mean ± SD (n = 6).

To this end, C57BL/6 mice were immunized with LZ901 on days 0 and 21, followed by subcutaneous implantation of B16 tumors on day 28. Selective depletion of immune cell populations was performed prior to therapy initiation, including pan‐B‐cell depletion, CD8^+^ T‐cell depletion, or NK‐cell depletion, using well‐established antibody‐mediated ablation strategies. On day 35, a single intratumoral dose of PBAP‐gE was administered, and tumor progression was monitored until study termination (Figure 3A).

Depletion of either B cells or NK cells markedly abrogated the antitumor efficacy of PBAP‐gE, whereas depletion of CD8^+^ T cells or treatment with an isotype control antibody had minimal impact on tumor control (Figure 3B,C). These results indicate that PBAP‐gE mediated tumor suppression is critically dependent on humoral immunity and innate effector function, rather than on virus‐specific cytotoxic T lymphocytes.

To further substantiate the role of humoral immunity, serum gE‐specific IgG titers were quantified longitudinally in mice from each treatment group. Notably, B cell depletion resulted in a sustained reduction in gE‐specific IgG levels, whereas no significant changes in antibody titers were observed in the other experimental groups over the same time course. These data confirm that B cells are the primary source of gE‐specific antibodies in this model and underscore the essential contribution of humoral immunity to PBAP‐gE–mediated antitumor activity (Figure 3D).

Longitudinal monitoring of body weight and multiplex serum cytokine profiling, further demonstrated that PBAP‐gE treatment did not elicit sustained systemic inflammation. In untreated mice, as well as in B cell‐ or NK cell‐depleted cohorts, progressive tumor growth was accompanied by slight body weight gain and unchanged or only marginally elevated inflammatory cytokine levels, including IL‐6, IFN‐γ, and TNF‐α. In contrast, mice subjected to CD8^+^ T cell depletion or treated with an isotype control antibody exhibited transient body weight loss and cytokine elevation coinciding with tumor regression, followed by rapid normalization once tumors were eradicated (Figure 3E). These results indicate that PBAP‐gE achieves effective tumor control without inducing sustained inflammatory toxicity.

Consistent results were obtained using a B16 melanoma model stably expressing firefly luciferase, in which tumor burden was monitored by longitudinal bioluminescence imaging. In this setting, B‐cell depletion abolished PBAP‐gE mediated tumor suppression, whereas CD8^+^ T‐cell depletion had no discernible effect (Figure S8A,B). Together, these findings establish vaccine‐induced antibodies, acting in concert with NK cells, as the dominant effectors of PBAP‐gE–driven antitumor activity.

### 2.4 Antitumor Efficacy of PBAP‐gE Strongly Correlates With Vaccine‐Induced Antibody Titers

**FIGURE 4:**
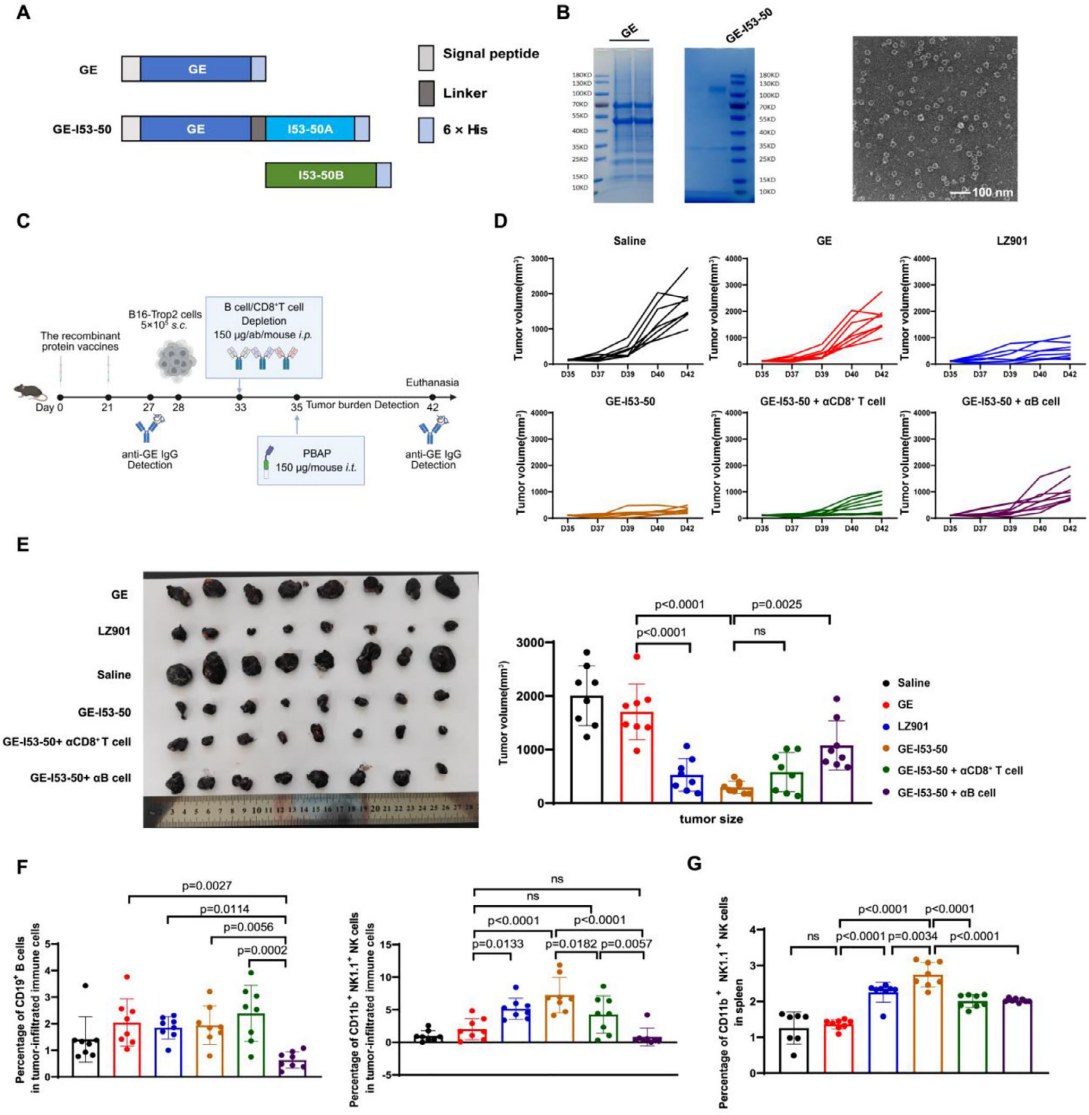
Antibody Titiers Dominate PBAP Antitumor Activity. (A) Schematic representation of the other two recombinant protein vaccines used in the study: the low‐immunogenicity gE subunit vaccine and the high‐immunogenicity GE‐I53‐50 virus‐like particle (VLP) vaccine formulated by I53‐50B and GE‐I53‐50A. (B) Coomassie Blue staining of gE and GE‐I53‐50 nanoparticles confirming the expression and purity of the recombinant proteins (left panel). Transmission electron microscopy (TEM) image showing the morphology of GE‐I53‐50 nanoparticles (right panel). Scale bars, 100 nm. (C) Overview of Experimental Design. C57BL/6J mice (8 mice per group) were immunized with the vaccine (5 µg per dose) on days 0 and 21. Serum samples collected on day 27 were analyzed to determine anti‐gE IgG titers. On day 28, 5 × 10^5^ B16‐Trop2 cells were subcutaneously implanted to establish tumor xenografts. On day 33, B cell depletion (via intraperitoneal injection of 150 µg/mouse each of anti‐CD19, anti‐CD22, and anti‐B220 antibodies) and CD8^+^ T cell depletion (via intraperitoneal injection of 150 µg/mouse anti‐CD8 antibody) were performed. On day 35, 150 µg/mouse PBAP‐gE was administered intratumorally, and tumor progression was monitored thereafter. Tumor volumes were measured every 1–2 days until day 42, at which point mice were euthanized for assessments of tumor burden and serum anti‐gE IgG levels. Created with BioRender.com. (D) Tumor growth curves were plotted for the six experimental groups: saline control, gE subunit vaccine, LZ901 vaccine, GE‐I53‐50 VLP vaccine, GE‐I53‐50 VLP + αCD8^+^ T cell depletion, and GE‐I53‐50 VLP + αB cell depletion. Corresponding anti‐gE‐specific IgG antibody levels are shown on the right, illustrating the correlation between vaccine‐induced antibody responses and tumor growth suppression. (E) Representative tumor images are shown on the left, with tumor volumes at the experimental endpoint shown in the right panel. The GE‐I53‐50 VLP vaccine groups showed significant tumor regression. Data are presented as the mean ± SD (n = 8). Statistical significance was determined using one‐way ANOVA. ns indicates not significant (p > 0.05). (F) Analysis of tumor‐infiltrating immune cells by flow cytometry, revealed that NK cells frequencies were significantly elevated in the tumor tissue of mice vaccinated with LZ901 or GE‐I53‐50 VLP, treated with PBAP‐gE. Data are presented as the mean ± SD (n = 8). Statistical significance was determined using one‐way ANOVA. ns indicates not significant (p > 0.05). (G) Analysis of NK cells in the spleen by flow cytometry revealed that NK cells frequencies were significantly elevated in the spleen of mice vaccinated with LZ901 or GE‐I53‐50 VLP, treated with PBAP‐gE. Data are presented as the mean ± SD (n = 8). Statistical significance was determined using one‐way ANOVA. ns indicates not significant (p > 0.05).

In our previous research, we developed a series of nanoparticle‐based SARS‐CoV‐2 vaccines that demonstrated significantly enhanced immunogenicity, inducing potent and durable immune responses [51, 52, 53, 54]. To further examine the relationship between endogenous antibody levels and PBAP‐gE efficacy, we evaluated PBAP‐gE mediated tumor suppression in conjunction with vaccines of varying immunogenicity. Mice were immunized with one of three gE‐based vaccine platforms: a low‐immunogenicity gE subunit vaccine, the clinically validated LZ901 vaccine, or a highly immunogenic nanoparticle‐based GE‐I53‐50 virus‐like particle (VLP) vaccine, which is self‐assembled from I53‐50B and GE‐I53‐50A (Figures 4A,B; Figure S9A) [55].

Following immunization and tumor establishment, selective immune cell depletion was performed on day 33 and PBAP‐gE was administered intratumorally on day 35, (Figure 4C). Both the LZ901 and GE‐I53‐50 VLP vaccines conferred strong antitumor activity when combined with PBAP‐gE, whereas the low‐immunogenicity subunit vaccine elicited only modest tumor control. Notably, depletion of B cells, but not CD8^+^ T cells, significantly diminished therapeutic efficacy in the GE‐I53‐50 VLP group, further reinforcing the central role of humoral immunity (Figure 4D,E).

**FIGURE 5:**
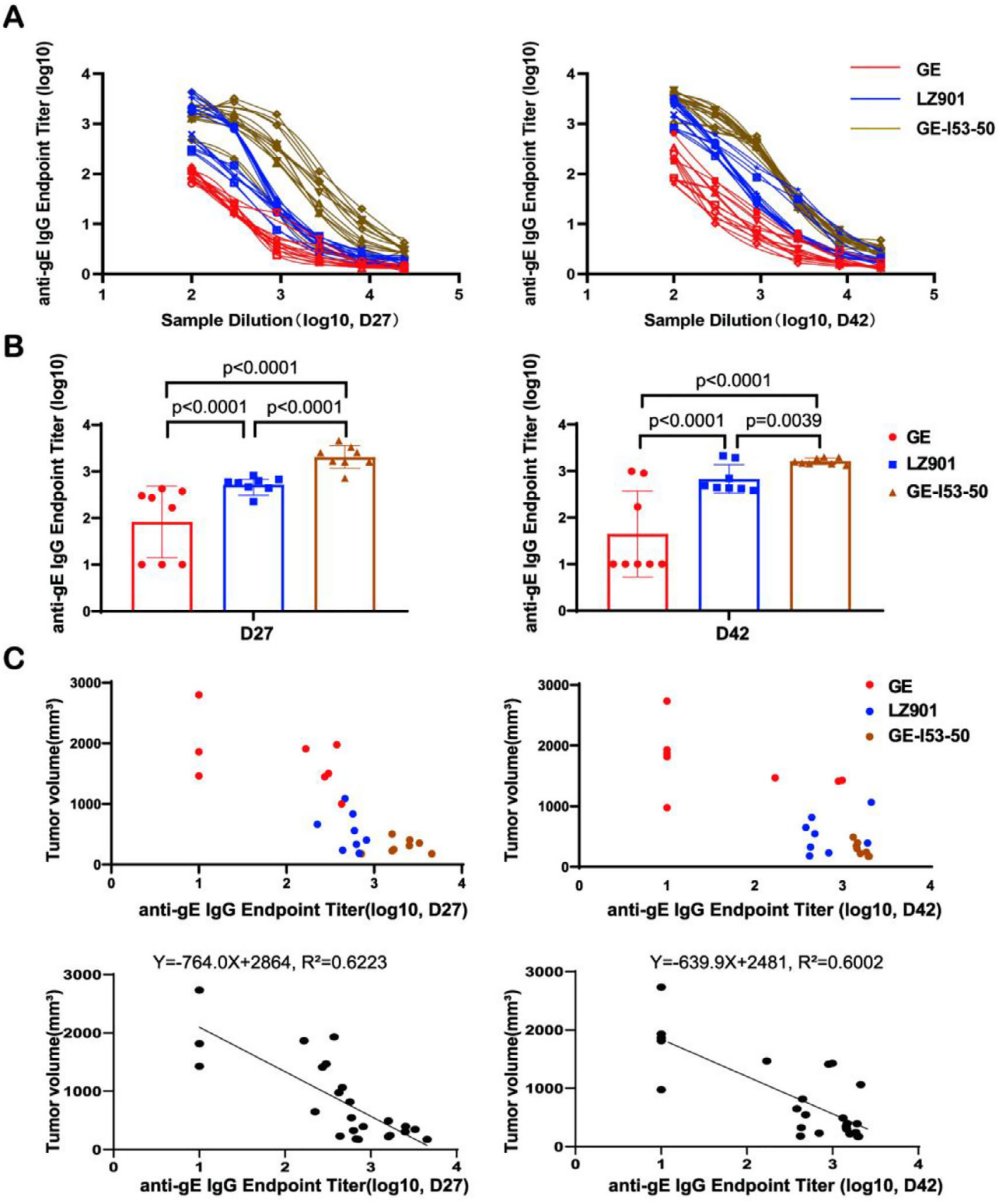
Tumor Burden is Inversely Correlated with Endpoint Titer of gE‐specific IgG Antibody Levels Across gE Subunit Vaccine, LZ901 Vaccine, and GE‐I53‐50 VLP Vaccine Treatment Groups. (A) The binding affinity of serum from LZ901‐vaccinated mice with gE was analyzed by ELISA among gE subunit vaccine, LZ901 vaccine, and GE‐I53‐50 VLP vaccine treatment groups. Data are presented as the mean ± SD, (n = 8). (B) The serum gE‐specific IgG antibody levels (Endpoint titer) at days 27 and 42 in mice immunized with the gE subunit vaccine, LZ901 vaccine, and GE‐I53‐50 VLP vaccine. Data are presented as the mean ± SD, (n = 8). Statistical significance was determined using one‐way ANOVA. Statistically significant differences were observed (p < 0.05). (C) Correlation analysis of tumor burden with serum gE‐specific IgG antibody levels (Endpoint titer) at days 27 and 42 in mice immunized with the gE subunit vaccine, LZ901 vaccine, and GE‐I53‐50 VLP vaccine.

Immunophenotypic analysis revealed no significant differences in CD4^+^ or CD8^+^ T cell infiltration across treatment groups (Figure S9B,C). In contrast, NK cell frequencies were significantly elevated in both tumor and spleen tissues of mice immunized with either LZ901 or the GE‐I53‐50 VLP vaccine (Figure 4F). Importantly, serum gE‐specific IgG titers at the experimental endpoint inversely correlated with tumor volume (Figure 5A–C; Figure S9D), demonstrating a strong quantitative relationship between antibody abundance and therapeutic outcome.

Collectively, these results establish vaccine‐induced antibody titers as a key determinant of PBAP‐gE mediated antitumor efficacy and underscore the importance of pairing PBAP‐based therapies with highly immunogenic vaccines.

### 2.5 PBAP Engineered With Tumor‐Specific Antigens Enable in Vitro Antitumor Activity With Therapeutic Antibodies and ADCs

**FIGURE 6:**
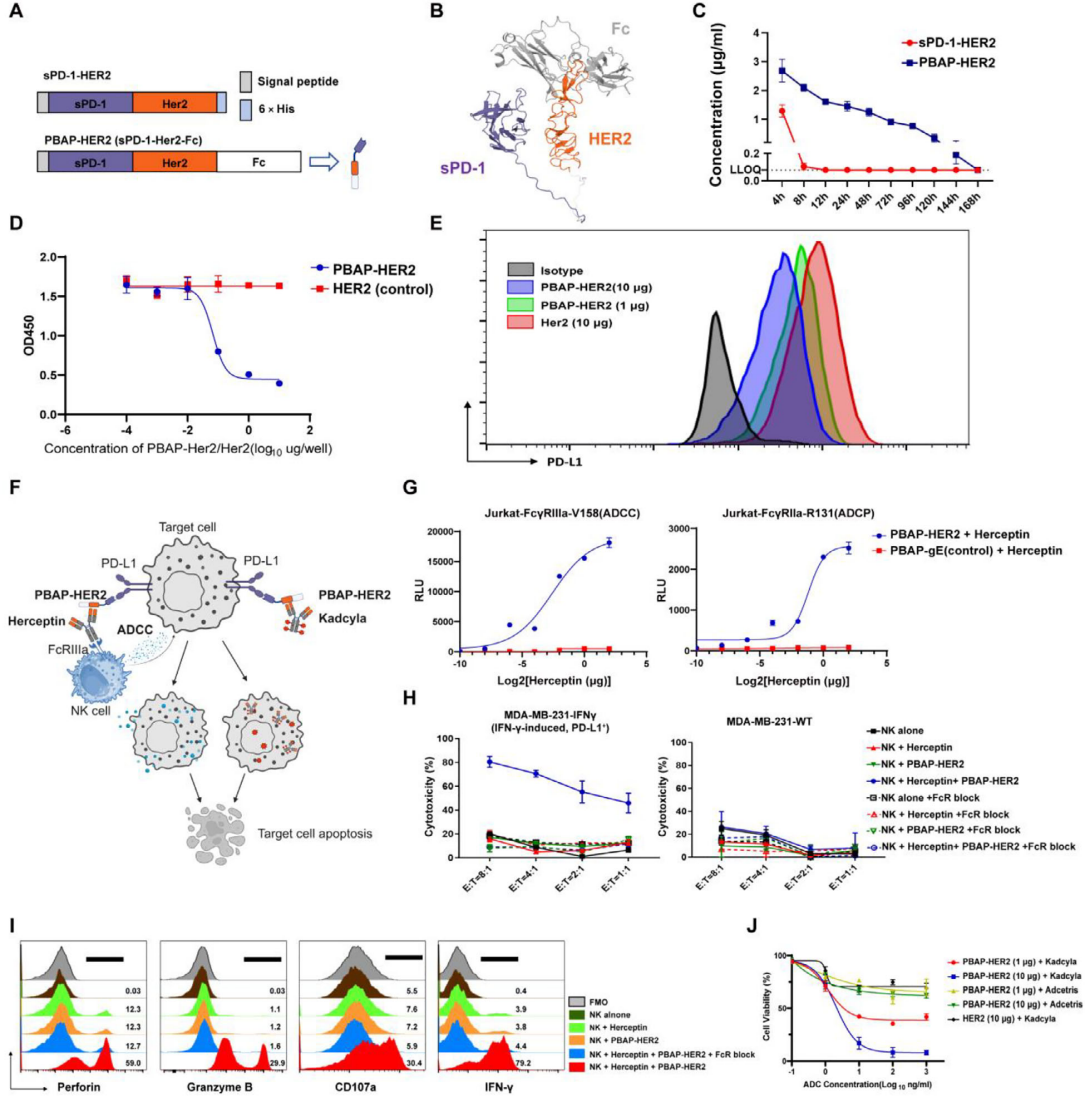
PBAP Conjugated with Tumor‐Specific Antigens Enhances Synergistic Anti‐Tumor Activity When Combined with Clinical Antibodies and Antibody‐Drug Conjugates (ADCs) In Vitro. A) Schematic representation of the design of sPD‐1‐HER2 and PBAP‐HER2 (sPD‐1‐HER2‐Fc). PBAP‐HER2 was engineered via the fusion of extracellular domain of human PD‐1 (sPD‐1) with Domain *IV* of HER2 protein, followed by the incorporation of an Fc region to enhance protein stability and prolong in vivo half‐life. vB) Structural modeling of PBAP‐HER2 with AlphaFold 3. (C) Pharmacokinetic profiles of sPD‐1‐HER2 and PBAP‐HER2 following intravenous injection into C57BL/6J mice (n=3 mice/group, 100 µg/mice). Data are presented as the mean ± SD (n = 3). (D) The binding inhibition of PBAP‐HER2 on PD‐L1/PD‐1 interaction was assessed by ELISA. The absorbance was measured at 450 nm to determine the blocking effect. Data are presented as the mean ± SD (n = 3). (E) The fluorescence intensity of the antibody‐cell binding was analyzed using a flow cytometry to assess the blocking effect on the PD‐1/PD‐L1 pathway. (F) Diagram illustrating the mechanism by which PBAP‐HER2 synergizes with Herceptin and Kadcyla to kill PD‐L1‐positive target cells. Created with BioRender.com. (G) ADCC and ADCP activities were assessed using Jurkat‐FcγR reporter systems: ADCC (FcγRIIIa‐V158 variant) and ADCP (FcγRIIa‐R131 variant) in response to PBAP‐HER2/PBAP‐gE combined with Herceptin. PBAP‐HER2 in combination with Herceptin significantly enhanced ADCC and ADCP activities against HER2‐negative MDA‐MB‐231 cells. Representative of 3 independent experiments. Data are presented as mean ± SD (n = 3). (H) NK cells were co‐incubated with PBAP‐Her2 and Herceptin, against MDA‐MB‐231‐IFN‐γ (IFN‐γ induced, PD‐L1^+^) tumor cells and MDA‐MB‐231‐WT cells. NK cells, NK cells co‐incubated with PBAP‐Her2, NK cells co‐incubated with Herceptin, and all groups treated with anti‐FcγRIII blocking antibody were used as controls. Data are presented as mean ± SD of 3 independent experiments, each performed in triplicate. (I) Flow cytometry analysis of perforin, granzyme B, IFN‐γ and CD107a in NK cells. Representative of 3 independent experiments. (J) The CCK8 assay was used to evaluate the cytotoxicity of commercial ADCs (Kadcyla and Adcetris) combined with PBAP‐HER2. MDA‐MB‐231‐PD‐L1‐OE cells were treated with PBAP‐HER2 (10 µg/well) for 4 h, followed by ADC drugs (Kadcyla or Adcetris) at various concentrations (0.1, 1, 10, 100, 1000 ng/mL). After 24 h of incubation, cell viability was measured using the CCK8 assay. PBAP‐HER2 with Adcetris and HER2 protein with Kadcyla were used as controls. Representative of 3 independent experiments. Data are presented as mean ± SD (n = 3).

To explore the versatility of the PBAP platform beyond viral antigens, we engineered a HER2‐targeted PBAP construct (PBAP‐HER2) by fusing the extracellular domain of human PD‐1 to Domain *IV* of HER2, followed by incorporation of an Fc domain to enhance stability (Figure 6A; Figure S10A, B). Structural modeling using AlphaFold 3 revealed no steric hindrance between functional domains (Figure 6B), and pharmacokinetic analysis demonstrated markedly prolonged in vivo persistence of PBAP‐HER2 compared with sPD‐1‐HER2 lacking the Fc domain (Figure 6C). In vitro experimental verification has demonstrated that PBAP‐HER2 inhibits the binding of PD‐L1 protein to PD‐L1 antibodies. Additionally, PBAP‐HER2 effectively competitively interferes with PD‐L1 antibody binding MDA‐MB‐231 cells engineered to stably overexpress murine PD‐L1 (designated MDA‐MB‐231‐PD‐L1‐OE) (Figures 6D,E).

This dual‐functional construct is designed to engage PD‐L1 on tumor cells via the sPD‐1 domain, while concurrently being recognized by HER2‐targeting monoclonal antibody trastuzumab (Herceptin) and the antibody‐drug conjugate Kadcyla, which combines Herceptin with a potent cytotoxic agent. The human‐derived MDA‐MB‐231‐PD‐L1‐OE tumor model, a HER2‐negative breast cancer cell line engineered to stably overexpress human PD‐L1 via lentiviral transduction and subsequent enrichment, were introduced to explore the potential synergistic effects with Herceptin and Kadcyla (Figure 6F).

To assess the potential of PBAP in synergy with Herceptin for mediating ADCC and antibody‐dependent cellular phagocytosis (ADCP), we employed two distinct reporter assays using Jurkat‐FcγRIIIa and Jurkat‐FcγRIIa cells, each expressing their respective Fc receptors and an NFAT‐driven luciferase reporter. In these assays, PBAP‐HER2 in combination with Herceptin generated robust ADCC signals, alongside moderate ADCP activity, in HER2‐negative MDA‐MB‐231‐PD‐L1‐OE cells (Figure 6G).

LDH‐release assays revealed that NK cells, when combined with PBAP‐HER2 and Herceptin, efficiently triggered ADCC against both constitutively PD‐L1‐overexpressing MDA‐MB‐231‐PD‐L1‐OE cells and IFN‐γ‐primed MDA‐MB‐231‐IFNγ (IFN‐γ‐induced, PD‐L1^+^) cells, markedly higher than the cytotoxicity observed against parental MDA‐MB‐231‐WT or CRISPR‐mediated PD‐L1‐knockout MDA‐MB‐231‐PD‐L1‐KO controls. (Figure 6H; Figure S11A–C). Importantly, saturating Fcγ receptors with a blocking antibody completely abolished cytotoxicity, formally proving that PBAP‐HER2 drives Herceptin‐directed, NK‐cell‐mediated ADCC strictly through PD‐L1‐dependent antibody bridging and not via any direct tumoricidal activity. Collectively, these data establish an obligate requirement for PD‐L1 engagement and underscore the potent synergy between PBAP‐HER2 and Herceptin in redirecting NK cells to eliminate tumor cells.

Flow‐cytometric profiling revealed that NK cells exposed to PBAP‐HER2 plus Herceptin simultaneously up‐regulated perforin, granzyme B and IFN‐γ, while exhibiting a higher frequency of CD107a, indicating a shift toward a highly activated and cytotoxic state (Figure 6I; Figure S12).

Finally, to further investigate the potential synergistic effect of PBAP‐HER2 with ADCs, we evaluated the combinatorial cytotoxicity of PBAP‐HER2 in combination with Kadcyla, using brentuximab vedotin (Adcetris, a CD30‐targeting ADC) as a negative control. The inclusion of PBAP‐HER2 significantly enhanced the cytotoxic activity of Kadcyla against MDA‐MB‐231‐PD‐L1‐OE cells, as determined by WST‐8 viability assay, with a marked reduction in cell viability compared to either treatment alone (Figure 6J). These findings demonstrate that PBAP‐HER2 can function as an antibody‐bridging adaptor, enabling therapeutic antibodies and ADCs to eliminate antigen‐negative tumors in a PD‐L1 dependent manner.

### 2.6 PBAP‐HER2 Combined With ADCs Demonstrates in Vivo Efficacy in NSG Tumor‐Bearing Mice

**FIGURE 7:**
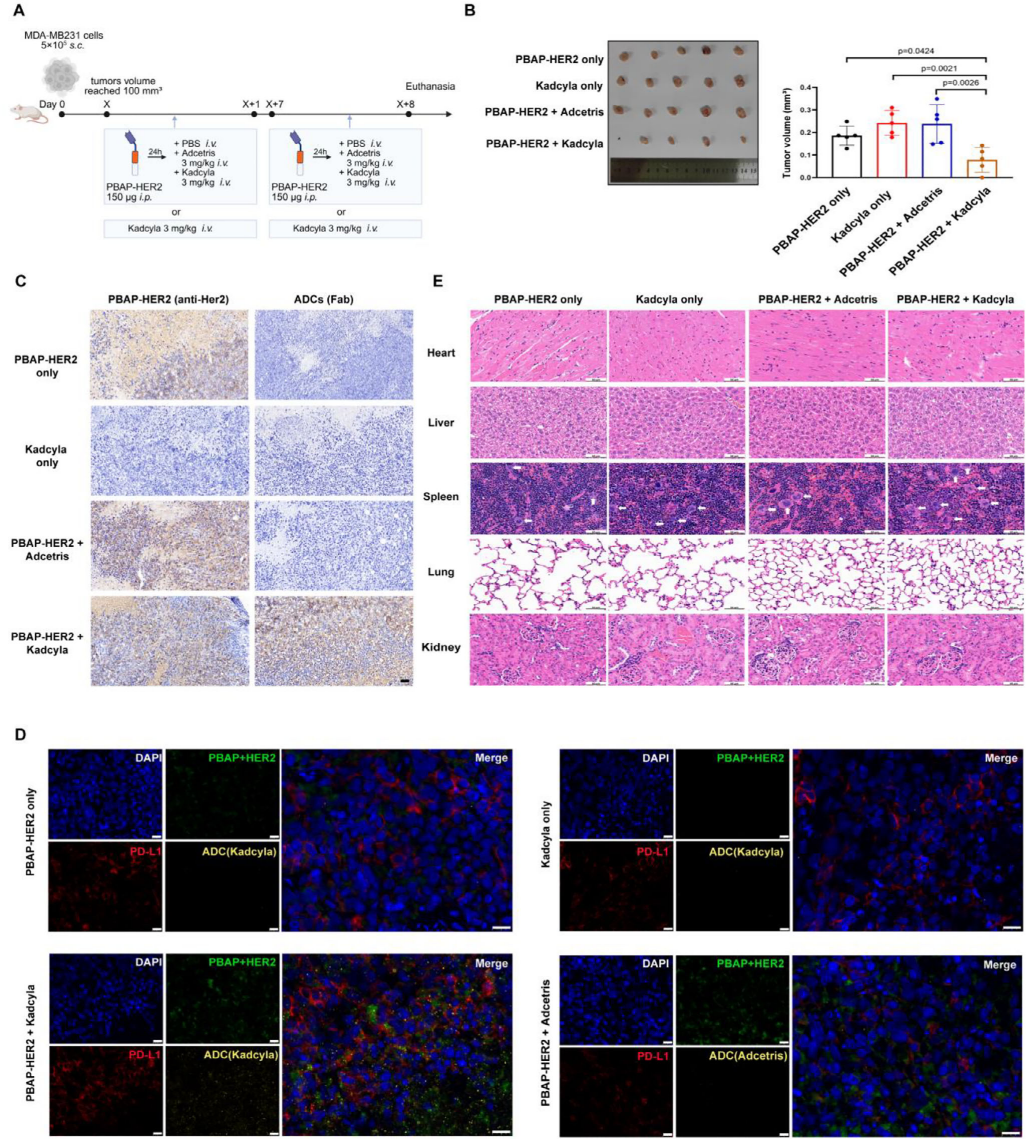
PBAP‐HER2 Synergizes with Antibody‐Drug Conjugates to Enhance Anti‐tumor Efficacy in NSG Mice Bearing Subcutaneous Tumors. (A) Overview of Experimental Design. NSG mice were subcutaneously inoculated with MDA‐MB‐231 cells. Once the tumors reached approximately 100 mm^3^, mice were assigned to one of four treatment groups (n=5 mice/group): PBAP‐HER2 alone, Kadcyla alone, PBAP‐HER2 + Adcetris, and PBAP‐HER2 + Kadcyla. PBAP‐HER2 (150 µg/mouse) was administered intraperitoneally, followed by tail vein injections of Kadcyla (3 mg/kg) or Adcetris (3 mg/kg) 24 h later. Treatments were administered once a week for two consecutive cycles, with tumor growth monitored throughout the study. At the experimental endpoint, mice were euthanized, and tumor and tissue samples were collected for further analysis. Created with BioRender.com. (B) Representative tumor images for each experimental group are displayed in the left panel, with tumor volumes at the experimental endpoint shown in the right panel. Notably, the PBAP‐HER2 + Kadcyla group exhibited the most significant tumor regression, accompanied by robust tumor control, relative to all other groups. Data are presented as the mean ± SD (n = 5). Statistical significance was determined using one‐way ANOVA. Statistically significant differences were observed (p < 0.05). (C) Immunohistochemistry (IHC) analysis was performed to evaluate the intratumoral infiltration of PBAP‐HER2 and ADC drugs. The results demonstrated that PBAP‐HER2 effectively infiltrated the tumor tissue. Furthermore, Kadcyla was found to exhibit intratumoral infiltration exclusively when co‐administered with PBAP‐HER2. In contrast, Adcetris failed to infiltrate tumor tissues in the PBAP‐HER2 plus Adcetris combination group. Scale bars, 50 µm. (D) Immunofluorescence analysis further confirmed the specific efficacy of the PBAP‐HER2 + Kadcyla combination. Tumor sections revealed clear co‐localization of PBAP‐HER2 with PD‐L1 on tumor cells. Kadcyla was observed to enter tumor cells exclusively in the PBAP‐HER2 + Kadcyla group. In contrast, no intracellular ADC uptake was detected in the control groups (PBAP‐HER2 + Adcetris or Kadcyla only). Scale bars, 20 µm. (E) H&E staining showed no significant histopathological damage to major organs (heart, liver, spleen, lungs) in the experimental group, indicating a favorable safety profile. An increased presence of multinucleated giant cells was observed in the spleens, particularly in the PBAP‐HER2 + Kadcyla group, as indicated by white arrows. Scale bars, 60 µm.

The in vivo efficacy of PBAP‐HER2 was next evaluated in NSG mice bearing subcutaneous MDA‐MB‐231 tumors. Once tumors reached approximately 100 mm^3^, mice were treated with PBAP‐HER2 alone, Kadcyla alone, PBAP‐HER2 plus Adcetris, or PBAP‐HER2 plus Kadcyla (Figure 7A).

Only the combination of PBAP‐HER2 and Kadcyla resulted in significant tumor regression, whereas all control groups exhibited continued tumor growth (Figure 7B). Immunohistochemistry (IHC) results demonstrated that PBAP‐HER2 specifically infiltrated tumor tissues. Notably, significant ADC drug infiltration within the tumor tissue was only observed in the PBAP‐HER2 + Kadcyla treatment group, indicating a synergy effect between PBAP‐HER2 and the ADC treatment in promoting targeted delivery (Figure 7C). Immunofluorescence analysis further confirmed that only the PBAP‐HER2 + Kadcyla group exhibited notable tumor regression or control. Tumor sections revealed clear co‐localization of PBAP‐HER2 with PD‐L1 on tumor cells. Furthermore, Kadcyla was observed to enter tumor cells exclusively in the PBAP‐HER2 + Kadcyla group, where it was internalized by the tumor cells, whereas no intracellular ADCs uptake was detected in the control groups, which included Kadcyla monotherapy or the PBAP‐HER2 + Adcetris combination therapy group (PBAP‐HER2 + Adcetris). This selective intracellular uptake highlights the enhanced targeting and delivery of the ADC, facilitated by PBAP‐HER2‐mediated tumor cell engagement (Figure 7D; Figure S13).

Hematoxylin and eosin (H&E) staining results, reviewed by two independent pathologists, revealed no significant histopathological damage to major organs (heart, liver, spleen, and lungs) in the experimental group, indicating a favorable safety profile. However, an increase in multinucleated giant cells was observed in the spleens, with particularly pronounced accumulation in the PBAP‐HER2 + Kadcyla group, suggesting a potential immune response or inflammatory process associated with the experimental treatment. This finding warrants further investigation into potential renal effects and the role of PD‐L1 expression in mediating off‐target responses (Figure 7E). In summary, these findings underscore the potent antitumor efficacy of PBAP‐HER2 in combination with ADCs, showing the capability to effectively eradicate solid tumors.

## 3 Discussion

Overcoming tumor immune evasion remains a fundamental challenge in cancer immunotherapy. A major limitation of current approaches is the frequent loss or downregulation of tumor‐specific antigens, which impairs CTLs recognition and constrains the efficacy of targeted therapies. To address this barrier, we developed a modular immune‐bridging strategy using PBAP to harness pre‐existing antiviral humoral immunity for tumor eradication. This approach selectively decorates PD‐L1 expressing tumor cells with non‐tumor viral antigens that are readily recognized by antibodies induced through prior vaccination or natural viral exposure. Given the widespread expression of PD‐L1 across major cancer types—including solid tumors as well as hematologic malignancies—our system offers a tumor‐agnostic, broadly applicable strategy.

This “tumor tagging” mechanism achieves dual functionality: it competitively disrupts PD‐1 inhibitory signaling while simultaneously redirecting antibody‐dependent effector functions, predominantly via NK cell–mediated ADCC, into the tumor microenvironment. Unlike conventional antigen‐targeting therapies, PBAP circumvents the constraints of tumor heterogeneity and antigen loss, providing a flexible platform applicable across diverse tumor types.

Focusing on PBAP‐gE, we demonstrated potent synergy with vaccine‐elicited antibodies and CAR‐T cell–mediated delivery, resulting in robust antitumor responses both in vitro and in vivo. Mechanistic studies revealed that the antitumor efficacy relied primarily on vaccine‐induced antibodies rather than CD8^+^ T cells, as confirmed through selective depletion experiments. Importantly, this immune‐bridging approach achieves tumor control without sustained systemic inflammation, underscoring its safety profile relative to oncolytic virus or bacterial antigen delivery systems. Moreover, CAR‐T–mediated, activation‐dependent PBAP expression provides pharmacological precision and minimizes systemic exposure, further enhancing therapeutic safety and efficacy.

From a translational standpoint, CAR‐T cells co‐expressing PBAP represent a promising fourth‐generation platform for rapid clinical development. NFAT‐driven expression links PBAP production directly to CAR‐T cell activation, ensuring localized delivery at tumor sites. Future strategies could incorporate tumor‐specific promoters, such as NR4A2 or RGS16, selectively active in tumor‐infiltrating T cells, to achieve spatially restricted PBAP expression and further reduce off‐target effects [56]. This approach positions PBAP as a versatile adjunct to therapies leveraging pre‐existing antiviral immunity, including licensed vaccines and potentially personalized vaccination strategies.

Beyond PD‐L1, PBAP constructs could be engineered to target additional immune‐suppressive ligands (e.g., CD155, B7‐H3, SIRPα) or tumor‐associated glycans, expanding its applicability to diverse tumor phenotypes and resistance mechanisms. Collectively, these findings establish PBAP as a highly flexible, tumor‐agnostic immune‐bridging platform with significant translational potential, capable of integrating humoral immunity, cellular therapy, and targeted delivery to overcome longstanding barriers in cancer immunotherapy.

## 4 Conclusion

PBAP establishes a flexible, tumor‐agnostic platform capable of targeting PD‐L1 to harness vaccine‐induced humoral immunity and amplify antibody‐mediated antitumor responses. By circumventing tumor antigen loss and intratumoral heterogeneity, PBAP expands the therapeutic landscape of cancer immunotherapy and provides a conceptual and translational framework for the development of next‐generation immune‐redirection strategies.

## 5 Experimental Section

### 5.1 Ethics Statements

The Ethics Review Board of Shenzhen Hospital of Southern Medical University (SHSMU) and Shenzhen TOP Biotechnology Co., Ltd (TOP) approved this study. Animal experiments were carried out in strict compliance with the guidelines and approved by Ethics Committee of SHSMU and TOP on Laboratory Animal Care (Assurance Number: 20240030, TOP‐IACUC‐2025‐0092).

### 5.2 Cell Lines and Culture Conditions

The human cancer cell lines of MDA‐MB‐231 (RRID: CVCL_0062), HEK 293T (RRID: CVCL_0063) and HEK 293F (RRID: CVCL_6642) were obtained from the American Type Culture Collection (ATCC). Mouse cell lines of 4T1 (RRID: CVCL_0125) and B16‐F10 (RRID: CVCL_0159) were obtained from the American Type Culture Collection (ATCC). Immortalized mouse NK cells (KIL C.2, RRID: CVCL_HC59) were obtained from Applied Biological Materials Inc. B16‐F10‐Trop2^+^ (B16‐Trop2) cells were established by infecting B16‐F10 with lentivirus carrying Trop2‐IRES‐GFP, followed by sorting GFP^high^ cells (BD FACS Aria II). B16‐F10‐fLuc (B16‐fLuc) were established by infecting B16‐F10 cells with lentiviruses carrying luciferase‐IRES‐RFP, followed by sorting RFP^high^ cells to enable bioluminescence *in vivo* imaging. MDA‐MB‐231, 293T, B16‐F10, B16‐F10‐Trop2^+^ (B16‐Trop2) and B16‐F10‐fLuc (B16‐fLuc) cells were cultured in DMEM medium (Invitrogen) supplemented with 10% FBS, 1% penicillin–streptomycin and 2 mM l‐glutamine (Invitrogen). HEK 293F cells were cultured in Union 293 medium (Union Bio) supplemented with 8 mM glutamine (ThermoFisher) and 1% penicillin‐streptomycin (ThermoFisher). 4T1 cells were cultured in RPMI‐1640 medium with 10% FBS, 2 mM l‐glutamine (Invitrogen) and 1% penicillin‐streptomycin (Invitrogen). KIL C.2 cells were cultured in PriGrow V medium (Applied Biological Materials) supplemented with 30% fetal bovine serum (Sigma Aldrich), 2 mM L‐glutamine, 50 ng/mL recombinant mouse stem cell factor (SCF, R&D Systems), 25 ng/mL recombinant mouse IL‐7 (R&D Systems) and 1% penicillin‐streptomycin (Invitrogen). 1 × 10^6^ cells/mL KIL C.2 cells were incubated with culture medium containing 20 ng/mL recombinant mouse IL‐2 (R&D Systems) overnight before use.

All cell lines except 293F cells were maintained in a humidified atmosphere containing 37°C and 5% CO_2_ and passaged two or three times a week. 293F cells were cultured at 37°C, 8% CO_2_ and 130 rpm speed in orbital shaker. All cell lines were authenticated by STR profiling and tested regularly for mycoplasma contamination using a MycoAlert Mycoplasma Detection kit (Lonza) and were only used when tested negative for contamination.

### 5.3 IFN‐γ‐induced PD‐L1 expression in 4T1 and MDA‐MB‐231 cells

4T1 cells were maintained in RPMI‐1640 medium (Invitrogen) supplemented with 10% FBS, 2 mM L‐glutamine and 1% penicillin‐streptomycin. MDA‐MB‐231 cells were cultured in DMEM medium (Invitrogen) containing 10% FBS, 2 mM L‐glutamine and 1% penicillin‐streptomycin. Both lines were incubated at 37°C under 5% CO_2_ and passaged two to three times per week. For PD‐L1 induction, 1 × 10^5^ cells were seeded per well of a 12‐well plate and allowed to adhere overnight. The medium was then replaced with fresh complete medium containing recombinant mouse IFN‐***γ*** (10 µg/mL, Sino Biological, 50709‐MNAH) for 4T1 cells or recombinant human IFN‐***γ*** (10 µg/mL, Sino Biological, 11725‐HNAS) for MDA‐MB‐231 cells. After 24 h stimulation, cells were harvested with 0.25% trypsin‐EDTA, washed twice with PBS, and subjected to flow‐cytometric validation.

### 5.4 Animal Models

Specific‐pathogen‐free (SPF) 6‐ to 8‐weeks‐old male C57BL/6 mice were purchased from Guangdong Medical Laboratory Animal Center (GDmLAC). Specific‐pathogen‐free (SPF) 6‐ to 8‐weeks‐old male NSG mice were purchased from Shanghai Model Organisms Center, Inc. Only male mice were used because the experimental design required repeated blood collections, and males generally tolerate serial bleeding better than females, thereby improving survival rates and minimizing animal loss. All mice were maintained in SPF barrier facilities at the Laboratory Animal Center of SHSMU and TOP, with free access to food and water. For ethical reasons, mice exhibiting >20% weight loss and cessation of food/water intake were promptly humanely euthanized as a humane endpoint.

### 5.5 Plasmid Construction

cDNA sequences of the target protein sPD‐1‐gE, GE, GE‐I53‐50A, sPD‐1‐HER2 with 6 × His‐tagged at C‐terminal and PBAP‐gE, PBAP‐HER2 were codon‐optimized and synthesized by Sangon Biotech into pcDNA3.1 plasmid. sPD‐1‐gE and PBAP‐gE were designed by fusing the extracellular domain of PD‐1 (sPD‐1) to viral antigen domain GE with a linker that was predicted and structurally optimized based on AlphaFold 3 modeling. PBAP‐gE was further engineered to incorporate an Fc domain (IgG2) and a signal peptide. Similarly, sPD‐1‐HER2 and PBAP‐HER2 were designed by fusing the soluble extracellular domain of PD‐1 (sPD‐1) to IV domain of HER2 using a optimized linker sequence and appending an Fc domain (IgG1, L234A, L235A) at the C‐terminus. In addition, PBAP‐HER2 was also engineered to include an Fc domain and a signal peptide. GE‐I53‐50A were designed by fusing the extracellular domain of VZV gE (aa 31‐544) to the I53‐50A component to enable antigen display on the corresponding NP carriers, with a signal peptide and 6 × His‐tag at C‐terminal. Competent Escherichia coli BL21(DE3) expressing protein‐related plasmid: DNA sequences of I53‐50B with 6 × His‐tagged at C‐terminal were synthesized by Sangon Biotech into pET28a vector.

Two anti‐Trop2 CAR constructs (Trop2‐CAR, Trop2‐CAR‐PBAP) and one control CAR construct (anti‐CD19) were synthesized and cloned into the third‐generation retrovirus plasmid backbones under the regulation of LTR promoter. All CAR constructs contained a mouse CD28 transmembrane and intracellular costimulatory domain in tandem with a mouse CD3ζ intracellular signaling domain. To construct a vector encoding PBAP‐gE in murine anti‐Trop2 CAR (Trop2‐CAR‐PBAP), the PBAP‐gE gene was inserted into the *BamHI* and *ClaI* restriction enzyme sites of the MIGR1 retroviral vector, and NFAT promoter was inserted between CAR and PBAP‐gE genes. The sequences of CD19 CAR were obtained from publicly available sequences for FMC63.

### 5.6 Protein Expression and Purification

Recombinant proteins sPD‐1‐gE, PBAP‐gE, GE, GE‐I53‐50A, sPD‐1‐HER2 and PBAP‐HER2 were heterologously produced in human embryonic kidney (HEK) 293F cells via transient plasmid transfection. Briefly, plasmids encoding the cDNA sequences of the corresponding target proteins were introduced into 293F cells using polyethyleneimine (PEI; Union Bio) at an optimal cell density of 2.0–3.0 × 10^6^ cells/mL. Subsequently, the transfected cells were maintained in suspension culture in Union 293 medium (Union Bio) under standardized conditions: 37°C, 8% CO_2_ atmosphere, and continuous orbital agitation at 130 rpm, with a 3‐4‐day incubation to allow maximal protein expression.

After the incubation period, cell culture supernatants were harvested and centrifuged to eliminate residual cellular debris. For purification of His‐tagged recombinant proteins (sPD‐1‐gE, GE, GE‐I53‐50A, sPD‐1‐HER2, PBAP‐HER2), the clarified supernatants were applied to Ni‐NTA affinity resin (Vazyme), and target proteins were eluted using a Tris‐HCl buffer containing a gradient (or fixed concentration) of imidazole. Given their distinct structural features, PBAP‐gE and PBAP‐HER2 underwent an additional purification step with HiTrap Protein A HP columns (Vazyme) to achieve higher homogeneity. All purified protein fractions were then concentrated and desalted via buffer exchange into Tris buffer. Protein concentrations were determined using a BCA protein assay kit, with bovine serum albumin (BSA) as the standard reference, while protein purity was assessed by sodium dodecyl sulfate‐polyacrylamide gel electrophoresis (SDS‐PAGE) followed by Coomassie Brilliant Blue staining.

I53‐50B was expressed and purified from BL21 *E.coli* (Biosharp) prokaryotic expression system induced by isopropyl‐D‐thiogalactopyranoside (IPTG, Biosharp). The bacterial cultures were harvested and lysed in Tris buffer (20 mM Tris, 50 mM NaCl, pH 7.5). After harvested and lysed by sonication, the supernatants were collected and incubated with Ni‐NTA agarose (Vazyme) to enrich His‐tagged I53‐50B, followed by protein elution with Imidazole ‐containing Tris buffer. The purified I53‐50B proteins were concentrated and buffer‐replaced with conventional Tris buffer. The concentration of I53‐50B was determined by BCA assay and Coomassie Blue staining were executed to confirm the purity.

For conjugation, I53‐50B and gE‐I53‐50A were mixed at a 3:1 mass ratio and incubated overnight at 2°C–8°C. Uncoupled gE‐I53‐50A and excessive I50‐53B were removed from the conjugated NP by size‐exclusion chromatography (SEC) using a Superdex 200 Increase 10/300 GL column (Cytiva) pre‐equilibrated with 20 mM Tris‐HCl, 25% sucrose w/v, pH 7.4 on the AKTA system (Cytiva). After separation, the conjugated gE‐I53‐50 nanoparticles were analysed on Coomassie Blue staining, western blotting, size‐exclusion chromatography (SEC), and transmission electron microscopy (TEM), to determine the coupling efficiency by densitometry as previously described. Besides, the bacterial endotoxins in nanoparticle were quantified by tachypleus amebocytelysate test (less than 100 EU/mg).

### 5.7 PBAP Inhibition of PD‐L1 Binding to PD‐1 in Vitro

ELISA Blocking Activity Detection of PBAP‐HER2: 96‐well plates were coated with 2 µg/mL human PD‐L1 (ECD, His Tag, SinoBiological, 10084‐H08H) protein in carbonate buffer overnight at 2°C–8°C. The next day, the plates were blocked with 2% BSA for 1 h at 37°C and then washed with 0.05% PBST. Subsequently, PBAP‐HER2 (10 µg/well) with a dilution ratio of 1:10, was added to the wells. 4 h later, PD‐L1 monoclonal antibody (SinoBiological, 10084‐MM33) was added to the coated plates at dilution of 1:10000, followed by incubation for 2 h at 37°C. After incubation, the plates were washed with 0.05% PBST and incubated with Goat anti‐Mouse IgG, HRP secondary antibody (SinoBiological, SSA007) for 1 h at 37°C. Finally, TMB substrate (SinoBiological, SEKCR01) was added and incubated for 10–15 min, followed by stopping reaction with stop solution (Solarbio) after sufficient development. The absorbance was measured at 450 nm using a microplate reader. The blocking effect of the antibody on PD‐1/PD‐L1 binding was determined based on the absorbance values.

For the ELISA blocking activity detection of PBAP‐gE, the same procedure was followed with the following modifications: human PD‐L1 (ECD, His Tag, SinoBiological, 10084‐H08H) protein was replaced with mouse PD‐L1 (ECD, His Tag, SinoBiological, 50010‐M08H), PBAP‐HER2 (1 µg/well and 10 µg/well) was replaced with PBAP‐gE (1 µg/well and 10 µg/well), PD‐L1 monoclonal antibody (SinoBiological, 10084‐MM33) was replaced with PD‐L1 monoclonal antibody (SinoBiological, B50010‐R678), and Goat anti‐Mouse IgG, HRP secondary antibody (SinoBiological, SSA007) was replaced with Goat anti‐Rabbit IgG, HRP secondary antibody (SinoBiological, SSA004). All other steps remained unchanged.

Flow cytometry Detection of PBAP‐HER2: MDA‐MB‐231‐PD‐L1 cells were plated in a 96‐well plate at a density of 1 × 10^5^ cells per well and maintained in a humidified atmosphere containing 37°C and 5% CO_2_ overnight. The next day, PBAP‐HER2 (10 µg/well) was added to the wells. 4 h later, PD‐L1 monoclonal antibody (SinoBiological, 10084‐MM33) was added to the coated plates, with a dilution of 1:5000, followed by incubation for 2 h, the cells were washed with PBS and incubated with PE‐labeled goat anti‐mouse IgG (H+L) fluorescent secondary antibody (Invitrogen, P‐852) for 30 min on ice. Finally, after another wash with PBS, the cells were analyzed using a flow cytometry to assess the blocking activity of the antibody on the PD‐1/PD‐L1 signaling pathway by measuring the fluorescence intensity of the antibody‐cell binding.

For the flow cytometry blocking activity detection of PBAP‐gE, the same procedure was followed with the following modifications: MDA‐MB‐231‐PD‐L1‐OE cells were replaced with 4T1‐PD‐L1‐OE cells, PBAP‐HER2 (10 µg/well) was replaced with PBAP‐gE (10 µg/well), PD‐L1 monoclonal antibody (SinoBiological, 10084‐MM33) was replaced with PD‐L1 monoclonal antibody (SinoBiological, B50010‐R678), PE‐labeled goat anti‐mouse IgG (H+L) fluorescent secondary antibody (Invitrogen, P‐852) was replaced with goat anti‐Rabbit IgG (H+L) fluorescent secondary antibody (Invitrogen, P‐2771MP). All other steps remained unchanged.

### 5.8 In Vitro ADCC Reporter Assay

The MDA‐MB‐231‐PD‐L1‐OE and MDA‐MB‐231‐PD‐L1‐KO cells were harvested and seeded into a white opaque 96‐well assay plate with 1.0 × 10^4^ cells in 100 µL of RPMI‐1640 medium with 2% FBS per well separately, followed by incubation at 37°C, 5% CO_2_ overnight (16–24 h). Subsequently, an overdosage of PBAP (10 µg/well) was added to the wells. 4 h later, trastuzumab (MCE, HY‐P9907) was serially diluted in the assay medium (RPMI‐1640 medium with 2% FBS) at a starting concentration of 4 µg/well, with a dilution ratio of 1:4. Jurkat‐FcγRIIIa (Vazyme, ADCC) and Jurkat‐FcγRIIa (Vazyme, ADCP) effector cells were then added to the plate at a density of 1.0 × 10^5^ cells per well. Jurkat‐FcγRIIIa (Vazyme, ADCC) and Jurkat‐FcγRIIa (Vazyme, ADCP) effector cells were then added to the plate at a density of 1.0 × 10^5^ cells per well. The plate was incubated at 37°C, 5% CO_2_ for 6 h. After incubation, 50 µL of Bio‐Lite reagent (Vazyme) was added to each well and relative luciferase units (RLU) were measured using the GloMax Navigator (Promega).

To further elucidate the effects of PBAP and trastuzumab on ADCC and ADCP activity, three control conditions were established: (1) no addition of PBAP, (2) no addition of trastuzumab, and (3) no addition of either PBAP or trastuzumab.

### 5.9 CRISPR Knockout of PD‐L1

CRISPR/Cas9 technology was used to knockout PD‐L1 in 4T1 cells and MDA‐MB‐231 cells. The sgRNA sequences targeting mouse (sgRNA: GTATGGCAG‐CAACGTCACGA) and human PD‐L1 (sgRNA: ACCGTTCAGCAAATGCCAGT)were designed using the online CRISPR design tool (Benchling) and transfected into 4T1 cells and MDA‐MB‐231 cells using Lipofectamine 3000 (Invitrogen) separately. Knockout cells were selected with puromycin (10 µg/mL) for 10 days and screened by flow cytometry for PD‐L1 negative populations.

### 5.10 Cytotoxicity Assays

The ability of KIL C.2 cells to kill tumor target cells via PBAP‐gE and LZ901 vaccined murine serum was measured by lactate dehydrogenase (LDH) assay. Briefly, The 4T1‐PD‐L1‐OE and 4T1‐PD‐L1‐KO cells were harvested and seeded into a transparent 96‐well assay plate (V bottom) with 1.0 × 10^4^ cells in 100 µL of PriGrow V medium with 30% FBS per well separately, followed by incubation at 37°C, 5% CO_2_ overnight (16–24 h). Subsequently, an overdosage of PBAP‐gE (10 µg/well) was added to the wells. 4 h later, LZ901‐vaccinated murine serum (Endpoint titer = 3.4) was diluted 100‐fold and then added to each well at a volume of 100 µL. KIL C.2 cells containing 20 ng/mL IL‐2 (R&D Systems) and IL‐7 (R&D Systems) were then added to the plate at a start density of 8.0 × 10^4^ cells/well, with a dilution ratio of 1:2. The plate was incubated at 37°C, 5% CO_2_ for 24 h.

To formally demonstrate that Fcγ‐receptor engagement is obligate for KIL C.2 cells mediated killing, TruStain FcX PLUS (BioLegend, 422301) antibody was used to saturate FcγR on the effector cells. KIL C.2 cells (8 × 10^4^ per well) were resuspended in 100 µL ice‐cold FACS buffer (PBS + 2 % FBS + 2 mM EDTA), incubated with 5 µg/mL TruStain FcX PLUS for 15 min at 4°C, and used immediately without washing. 4T1‐IFNγ (IFN‐γ induced, PD‐L1^+^) or 4T1‐WT cells were treated with PBAP‐gE and LZ901‐hyperimmune serum, and cytotoxicity was assessed by LDH release assay as described.

LDH release was measured using the CytoTox96 nonradioactive cytotoxicity assay (Promega) according to the manufacturer's instructions. Absorbance values from wells containing effector cells alone and target cells alone were detected and subtracted as background from the co‐culture values. Wells containing target cells alone were lysed with a lysis reagent for 30 min at 37°C, and the resulting luminescence was set as 100% lysis. Cytotoxicity was calculated using the following formula:

$$\begin{array}{ccc} & & \%\mathrm{Cytotoxicity}\\ & & =\frac{\left(\mathrm{Experimental}-\mathrm{Effector}\;\mathrm{spontaneous}-\mathrm{Target}\;\mathrm{spontaneous}\right)}{\left(\mathrm{Target}\;\mathrm{maximum}-\mathrm{Target}\;\mathrm{spontaneous}\right)}\\ & & \;\times 100\% \end{array}$$

To evaluate the in vitro cytotoxicity of Trop2‐CAR‐PBAP cells against target cells, the B16‐Trop2 cells were harvested and seeded into a transparent 96‐well assay plate (V bottom) with 1.0 × 10^4^ cells in 100 µL of 1640 medium with 10% FBS per well separately, followed by incubation at 37°C, 5% CO_2_ overnight (16–24 h). Subsequently, Trop2‐CAR cells (negative control) and Trop2‐CAR‐PBAP cells containing 20 ng/mL IL‐2 (R&D Systems) were then added to the plate at a start density of 8.0 × 10^4^ cells/well, with a dilution ratio of 1:2. The plate was incubated at 37°C, 5% CO_2_ for 24 h. LDH release was measured using the CytoTox96 nonradioactive cytotoxicity assay (Promega) according to the manufacturer's instructions and cytotoxicity was calculated using the same formula as described above.

Similarly, the cytotoxic activity of NK cells against tumor target cells mediated by PBAP‐HER2 and trastuzumab was evaluated using the LDH assay. Briefly, the MDA‐MB‐231‐PD‐L1‐OE and MDA‐MB‐231‐PD‐L1‐KO cells were harvested and seeded into a transparent 96‐well assay plate (V bottom) with 1.0 × 10^4^ cells in 100 µL of RPMI‐1640 medium with 10% FBS per well separately, followed by incubation at 37°C, 5% CO_2_ overnight (16–24 h). Subsequently, an overdosage of PBAP‐HER2 (10 µg/well) was added to the wells. 4 h later, trastuzumab (1 µg/well) was added. NK cells were then added to the plate at a start density of 8.0 × 10^4^ cells/well, with a dilution ratio of 1:2. The plate was incubated at 37°C, 5% CO_2_ for 24 h. LDH release was measured using the CytoTox96 nonradioactive cytotoxicity assay (Promega) according to the manufacturer's instructions and cytotoxicity was calculated using the same formula as described above.

To confirm that FcγR engagement is likewise required for human NK‐cell cytotoxicity, purified donor NK cells (8 × 10^4^ /well) were pre‐treated with 5 µg/mL Human TruStain FcX (Fc Receptor Blocking Solution) (BioLegend, 422301) in 100 µL ice‐cold FACS buffer (15 min, 4°C, no wash) before addition to PBAP‐HER2–coated MDA‐MB‐231‐IFN‐γ (IFN‐γ induced, PD‐L1^+^) or MDA‐MB‐231‐WT targets in the presence of trastuzumab; LDH release was quantified exactly as above.

To evaluate the in vitro cytotoxicity of ADC drugs (Kadcyla) in combination with PBAP‐HER2 against target cells, a CCK8 assay was performed. Briefly, the MDA‐MB‐231 cells were harvested and seeded into a transparent 96‐well assay plate (V bottom) with 1.0 × 10^4^ cells in 100 µL of RPMI‐1640 medium with 10% FBS per well separately, followed by incubation at 37°C, 5% CO_2_ overnight (16–24 h). Subsequently, an overdosage of PBAP‐HER2 (10 µg/well) was added to the wells. 4 h later, ADC drugs (Kadcyla or Adcetris) were added at various concentrations (0.1, 1, 10, 100, and 1000 ng/mL). The plates were incubated at 37°C with 5% CO_2_ for 24 h. Cytotoxicity was assessed using the CCK8 assay according to the manufacturer's instructions (Dojindo Molecular Technologies), with absorbance measured at 450 nm using a microplate reader. The percentage of cell viability was calculated relative to untreated control cells.

### 5.11 Negative Stain Electron Microscopy

Transmission electron microscopy (TEM) grids of gE‐I53‐50 nanoparticles were subjected to negative‐stain electron microscopy at Shenzhen Medical Academy of Research and Translation. Briefly, 3.5 µL of each sample (0.1 mg/mL) was applied to glow‐discharged TEM grids coated with a thin continuous carbon film and stained with 2% uranyl acetate. Imaging was performed using a Hitachi HT7800 microscope operating at an acceleration voltage of 120 kV. Images were recorded at a magnification of 80,000× and a defocus of 1.5 µm.

For sample preparation, gE‐I53‐50 nanoparticles were first diluted to 0.1 mg/mL in 20 mM Tris pH 7.0, 150 mM NaCl. Then, 3.5 µL of the sample was applied to freshly glow‐discharged 300‐mesh carbon grids. After incubation for 1 min, excess liquid was blotted away with filter paper (Whatman). The grids were then stained by applying 10 µL of 2% uranyl acetate, followed by blotting; this staining step was repeated three times, with the third staining incubated for 1 min. Subsequently, the grids were washed three times with 10 µL of ultrapure water, and excess liquid was blotted away. Finally, the grids were air‐dried for 1 min before imaging. Prepared grids were examined under a Hitachi HT7800 electron microscope at a magnification of 80,000×.

### 5.12 Dynamic Light Scattering

Dynamic Light Scattering (DLS) was used to measure hydrodynamic diameter (Dh) and % Polydispersity (%Pd) of gE‐I53‐50 nanoparticle samples on an UNcle Nano‐DSF (UNchained Laboratories). Sample was applied to a 8.8 µL quartz capillary cassette (UNi, UNchained Laboratories) and measured with 10 acquisitions of 5 s each, using auto‐attenuation of the laser. Increased viscosity due to 4.5% v/v glycerol in the gE‐I53‐50 nanoparticle buffer was accounted for by the UNcle Client software in Dh measurements.

### 5.13 Transfection and Virus Production

Retroviral vectors encoding Trop2‐CAR, Trop2‐CAR‐PBAP and CD19‐CAR were produced by co‐transfecting HEK‐293T cells with the packaging plasmids (PCLECO) separately. Transfection was carried out using a standard calcium phosphate precipitation method, following a protocol optimized for efficient virus production. After 48 h of incubation, viral supernatants were collected and filtered through a 0.45 µm filter to remove cell debris. To concentrate the lentivirus, the supernatants were subjected to ultracentrifugation at 25,000 × g for 2 h at 4°C, and the resultant viral pellet was resuspended in a small volume of phosphate‐buffered saline (PBS). The concentrated lentivirus was aliquoted and stored at −80°C for future use.

### 5.14 Mouse CAR‐T Cell Generation

To generate mouse CAR‐T cells, T cells were isolated from the spleen and lymph nodes of C57BL/6 mice using the mouse T cell isolation kit (STEMCELL Biotec). These isolated T cells were activated with immobilized anti‐CD3e antibody (5 µg/mL, eBioscience) and anti‐CD28 (2 µg/mL, eBioscience) antibodies in the presence of murine IL‐2 (ProSpec) in RPMI‐1640 medium containing 10% FBS, 1% penicillin‐streptomycin, and 55 nM β‐Mercaptoethanol. After 24 h activation, the T cells were transduced with retroviral supernatants at an optimized multiplicity of infection (MOI), and spinoculation was performed at 800 × g for 90 min at 32°C to enhance transduction efficiency. Following transduction, the cells were cultured and expanded in RPMI‐1640 medium with 10% FBS and murine IL‐2 (100 IU/mL) for 3 days. The CAR expression was determined with flow cytometry analysis, and the functionality of the CAR‐T cells was evaluated by cytotoxicity assays and cytokine release assays.

### 5.15 PBAP Secretion Assay of Trop2‐CAR‐PBAP

To evaluate the PBAP secretion of Trop2‐CAR‐PBAP cells, 1.0 × 10^6^ Trop2‐CAR cells (negative control) and Trop2‐CAR‐PBAP cells were aliquoted and cultured with B16‐F10 cells at a ratio of 2:1 under 1640 medium with 10% FBS. The supernatants of the culture medium were sampled after 48 h. To assess PBAP‐secreting capabilities after consistent antigen stimulation, 1.0 × 10^6^ Trop2‐CAR cells (negative control) and Trop2‐CAR‐PBAP cells were aliquoted and cultured with B16‐Trop2 cells at a ratio of 2:1 under 1640 medium with 10% FBS. The supernatants of the culture medium were sampled after 48 h. Concentrations of PBAP were determined by ELISA (His Tag ELISA Detection Kit, L00435, GenScript), under the instructions of the manufacturer.

### 5.16 Tumor Infiltrated B cell Separation and Identification

To isolate and culture B cells from B16‐Trop2 tumor tissue, tumors were collected in PBS and homogenized through 70 mm strainers, and incubated in ACK lysis buffer to remove red blood cells (RBCs), followed by centrifuging and passing through a 40 mm strainer to obtain single cells. To enrich for B cells, CD19‐MicroBeads were used (Miltenyi Biotec, 130‐121‐301), followed by magnetic cell sorting (MACS). The positively selected B cells are then cultured in RPMI‐1640 medium supplemented with 10% FBS, 1% penicillin‐streptomycin, and 50 µM β‐mercaptoethanol. 1.0 × 10^6^ cells are incubated at 37°C and 5% CO_2_. B cells coculture with 5 ng/mL IL‐4 (Beyotime, P5916) and 2 µg/mL CD40L (Biolegend, 797404) for antibody secretion.

### 5.17 In Vivo Syngeneic Tumor Models

For in vivo experiments testing PBAP‐gE delivered via CAR‐T cells or intratumoral injection against B16‐Trop2 tumor cells in murine tumor models, 6–8 week‐old male C57BL/6 mice were immunized with the recombinant zoster vaccine LZ901 on days 0 and 21 (5 µg/mouse/dose, *i.m*.). On day 27, sera were collected. On day 28, mice were subcutaneously inoculated with B16‐Trop2 cells. When average tumor volumes reached ∼100 mm^3^, treatments were administered as per the experimental design. Tumor sizes were meticulously monitored throughout the study period, and blood samples and tumor tissues were collected for in‐depth analysis when mice were euthanized.

To dissect whether PBAP‐gE tumor suppression is executed by vaccine‐induced antibodies (via NK‐cell ADCC) or by gE‐specific CD8^+^ T cells, we designed two independent, non‐overlapping mouse experiments.

Experiment 1: C57BL/6 mice (n = 6 per group) were immunized with the recombinant zoster vaccine LZ901 on days 0 and 21 (5 µg/mouse/dose, *i.m*.). On day 28, parental B16 cells (5 × 10^5^) were implanted subcutaneously in the right flank. On day 33, animals received a single intraperitoneal depletion cocktail: anti‐CD19, anti‐CD22 and anti‐B220 (150 µg each, clones 1D3, CY34.1, RA3‐6B2) to eliminate B cells; anti‐NK1.1 (clone PK136, 150 µg) to remove NK cells; or anti‐CD8 (clone 16‐0081‐85, 150 µg) to delete CD8^+^ T cells. On day 35, mice were injected intratumorally with PBAP‐gE (150 µg). Tumor dimensions and body weight were recorded every 2‐3 days with digital calipers; volume = (length × width [2])/2. On day 42, mice were euthanized for tumor collection.

Experiment 2: A separate cohort (n = 3–5 per group) was handled identically through day 35, except that B16‐fLuc cells (5 × 10^5^) were implanted on day 28 and only B‐cell or CD8^+^ T‐cell depletion was carried out (same antibodies and doses as above). Tumor growth was tracked longitudinally by bioluminescence (IVIS Spectrum, 150 mg/kg d‐luciferin *i.p*., 2 min post‐injection, 60‐s exposure) on days 34, 36, 38 and 40; radiance was quantified with Live Imaging Software (Perkin Elmer).

To further delineate the association between endogenous antibody levels and the therapeutic efficacy of PBAP‐based treatments, GE subunit vaccine and GE‐I53‐50 VLP vaccine were introduced. C57BL/6 mice were divided into six groups (n = 8 per group): saline control, GE subunit vaccine, LZ901 vaccine, GE‐I53‐50 VLP vaccine, and two GE‐I53‐50 VLP groups with either B cell or CD8^+^ T cell depletion. Vaccinations were administered on days 0 and 21 (5 µg/mouse/dose), with sera collected on day 27. Tumors were established by subcutaneous injection of B16‐Trop2 cells on day 28. Depleting antibodies were administered on day 33, and PBAP‐gE was injected intratumorally on day 35 (150 µg/mouse).

### 5.18 In Vivo Xenograft Models

NSG mice were subcutaneously inoculated with 5 × 10^6^ MDA‐MB‐231 cells (in 100 µL PBS). Mice were assigned to the following treatment groups: PBAP‐HER2 control, Kadcyla control, PBAP‐HER2 + Adcetris (CD30‐targeting ADC) control, and PBAP‐HER2 + Kadcyla. Tumor growth was monitored regularly, and when the average tumor volume reached approximately 100 mm^3^, treatments were initiated according to the experimental design. PBAP‐HER2 (150 µg/mouse) was administered intraperitoneally, followed 24 h later by Kadcyla (3 mg/kg) via tail vein injection. For the control groups, Adcetris (3 mg/kg) was administered via the same route and schedule. Both treatments were given once a week for two consecutive cycles. Mice were euthanized 7 days after the second ADC treatment for tissue and tumor collection. Upon euthanasia, tumor weights were measured, and the hearts, livers, spleens, lungs, kidneys, and tumor tissues were collected for subsequent in‐depth analysis.

### 5.19 Enzyme Linked Immunosorbent Assay (ELISA)

For test the anti‐gE IgG in blood samples and B cells of C57BL/6 mice, recombinant gE antigen was coated on high‐binding 96‐well plates at 2 µg/mL overnight at 2°C–8°C. After washing with 0.05% PBST, plates were blocked with 2% BSA in PBST for 1 h. Immunized mice serum were serially diluted and added into each well in duplicate followed by incubating at room temperature for 1 h. After washing with PBST, the detection of gE‐specific IgG antibody in serum of BALB/c was conducted through adding HRP‐conjugated goat anti‐mouse (SinoBiological, SSA007) respectively at dilution of 1:10000 and incubating for another 1 h. After washing with PBST, HRP substrate TMB solution (SinoBiological, SEKCR01) was added, followed by stopping reaction with stop solution (Solarbio) after sufficient development. Plates were immediately read at 450 nm and the data was analyzed using GraphPad Prism 8.0 software for non‐linear regression to calculate endpoint titers.

For serum cytokine quantification, the following Sino Biological ready‐to‐use ELISA kits were employed exactly as supplied: Mouse IL‐6 ELISA Kit (Cat. 50136A), Mouse IFN‐γ ELISA Kit (Cat. 50709A) and Mouse TNF‐α ELISA Kit (Cat. 50349A). Briefly, 50 µL of C57BL/6 mouse serum (diluted 1:2 in the provided Sample Diluent) was added in duplicate to the antibody‐pre‐coated 96‐well plates and incubated 1 h at 37°C. After three washes with 300 µL per well of the supplied Wash Buffer (0.05 % Tween‐20), 100 µL of biotinylated detection antibody was added for 45 min at 37°C. Plates were washed again, incubated with 100 µL HRP–streptavidin conjugate for 30 min at 37°C, and developed with 100 µL TMB Substrate Reagent for 10 min in the dark. The reaction was stopped with 50 µL Stop Solution and absorbance was read immediately at 450 nm (reference 630 nm). Cytokine concentrations were interpolated from the respective 7‐point standard curve (0‐1000 pg/mL) using GraphPad Prism 8.0.

### 5.20 Flow Cytometry

PD‐L1 surface expression was verified by staining 1 × 10^5^ cells with PE‐conjugated anti‐mouse PD‐L1 (BioLegend, 124307) or anti‐human PD‐L1 (BioLegend, 374511) for 30 min on ice. Cells were washed twice with PBS containing 0.5% BSA. Isotype‐matched antibodies served as negative controls.

For intracellular cytokine staining, lymphocytes were first surface‐labeled with FITC anti‐mouse NK1.1 (BioLegend, 156507) and APC anti‐mouse CD107a (BioLegend, 121613) for KIL.2 cells, or FITC anti‐human CD56 (BioLegend, 304603) and APC anti‐human CD107a (BioLegend, 301103) for human NK cells, Cells were then fixed and permeabilized with 1 mL Permeabilization Buffer (Invitrogen, 00‐8333‐56). KIL.2 cells were subsequently stained with PE anti‐mouse perforin (BioLegend, 154305), PE‐Cyanine7 anti‐mouse/human granzyme B (BioLegend, 396409), and SPARK NIR 685 anti‐mouse IFN‐γ (BioLegend, 505861). Human NK cells were stained with PE anti‐human perforin (BioLegend, 505829), PE‐Cyanine7 anti‐mouse/human granzyme B (BioLegend, 396409), and SPARK NIR 685 anti‐human IFN‐γ (BioLegend, 502551).

The spleens and tumors were collected in PBS and homogenized through 70 mm strainers, and incubated in ACK lysis buffer to remove red blood cells (RBCs), followed by centrifuging and passing through a 40 mm strainer to obtain single cells. For the staining of lymphocyte surface markers, cells were stained with indicated fluorochrome‐conjugated monoclonal antibodies for 30 min within PBS containing 0.5% BSA on ice. The following indicated antibodies were used: FITC anti‐GGGGS (Hycells, GS‐ARFT), FITC anti‐mouse CD90.2 (Biolegend, 105306), PE anti‐mouse CD4 (Biolegend, 100408), APC anti‐mouse CD8a (Biolegend, 100712), Pacific Blue anti‐mouse CD19 (Biolegend, 152416), PE‐Cyanine7 anti‐mouse CD11b (Biolegend, 101215), PE‐Dazzle 594 anti‐mouse NK1.1 (Biolegend, 108747).

All samples were analyzed by flow cytometry.

### 5.21 Histopathology and Immunohistochemistry

MDA‐MB‐231 cells xenograft NSG mice were euthanized and major tissues, included heart, liver, spleen, lung, kidney, were collected and fixed in 4% paraformaldehyde buffer for 48 h, followed by embedding with paraffin. Longitudinal sections were performed and the sections (3–4 µm) were stained with H&E. Images were captured with Digital Slide Scanner (3DHISTECH, Pannoramic MIDI).

Tumor sections (3 µm thick) were stained with HER2 specific antibody (Sinobiological, 310184‐T08, Rabbit PAb) to assess PBAP‐gE infiltration and anti‐human Fab (Abcam, ab771, Mouse Mab) to evaluate the penetration and localization of ADCs within the tumor tissue. IHC was performed with HRP‐conjugated secondary antibodies and visualized with DAB substrate (Vector Laboratories).

### 5.22 Immunofluorescence Staining and Imaging

Briefly, the tumors were excised and prepared using the Swiss roll technique, fixed with BD Cytoperm/Cytofix (BD Bioscience, 554722) solution (diluted with PBS at 1:2) overnight at 2°C–8°C, followed by dehydrated in 30% sucrose for 12‐16 h before embedding in OCT compound (Sakura Finetek, 4583). 8–10 µm sections were prepared by CRYOSTAR NX5 (Thermo). The experiment assessed PBAP‐gE infiltration and ADC localization within tumor tissue using three‐color fluorescence staining. Paraffin‐embedded tissue sections were deparaffinized, followed by antigen retrieval and endogenous peroxidase blocking. The sections were incubated with anti‐human PD‐L1 (Abcam, ab279293, Mouse Mab) to label PD‐L1 expression on tumor cells, HER2‐specific antibody (Sinobiological, 310184‐T08, Rabbit PAb) to detect PBAP‐gE infiltration, and anti‐human Fab (Abcam, ab771, Mouse Mab) to evaluate the penetration and localization of ADCs within the tumor tissue. Signal amplification was achieved using HRP‐conjugated secondary antibodies and iF488/555‐TSA working solution. DAPI was used for nuclear counterstaining, and autofluorescence was quenched. The sections were then mounted with anti‐fluorescence quenching mounting medium, and fluorescence microscopy was performed to observe and capture images. The experiment also included an antibody elution step to ensure signal specificity. Fluorescence three‐color staining was conducted using the Servicebio TSAPLus Fluorescent Triple‐Color Staining Kit (G1236).

### 5.23 Statistical Analysis

All experiments were performed in triplicates or more, and data are presented as the mean ± SD. Statistical significance was determined using one‐way ANOVA with Tukey's post‐test or unpaired t‐test or Kruskal‐Wallis test where appropriate. A p‐value of less than 0.05 was considered statistically significant.

## Author Contributions

F.Z. supervised the project; F.Z., W.W., S.S., and Y.X.designed the study; H.G., Lijuan Lu, X.X., Y.L., D.H., H.F., G.T., C.S., Z.Z, N.L., X.L., J.T., L.H., and T.L. conducted the experiments; H.G., Lijuan Lu, X.X., Y.L., D.H., W.F., C.L., H.F., G.T., C.S., Z.Z, N.L., X.L., J.T., L.H., and S.S. acquired data; F.Z., H.G., Lijuan Lu, W.F., X.X., Y.L., L.P., Lu Lu, and Y.Z. analyzed data; F.Z., H.G., L.L., W.F., X.X., Y.L., D.Z., Z.F., and Y.X. wrote the manuscript.

## Funding

This work was supported by Shenzhen Science and Technology Program (JCYJ20250604190324033) and Natural Science Foundation of China (82003252) to F.Z; Guangdong Basic and Applied Basic Research Foundation of China (2023A1515220144) to W.W; Natural Science Foundation of China (82202986) and Natural Science Foundation of Guangdong Province (2023A1515220017, 2025A1515010135) to J.T; Natural Science Foundation of Guangdong Province (2024A1515012867) and Doctor “Sailing” project of Science and Technology Department of Guangzhou (2024A04J3291) to T.L; Sanming Project of Medicine in Shenzhen (No. SZSM202311032) to L.H.

## Conflicts of Interest

F. Z., N.L., and X.L. are inventors on patents related to the use of PBAP. The remaining authors declare no competing financial interests.

## Supporting information




**Supporting File**: advs74209‐sup‐0001‐SuppMat.docx.

## Data Availability

The data that support the findings of this study are available from the corresponding author upon reasonable request.
