# Supplementary material for: PD‐L1‐Binding Antigen Presenters: Redirecting Vaccine‐Induced Antibodies for Cancer Immunotherapy
Source: Adv Sci (Weinh). 2026 Feb 11;13(20):e19574. doi: 10.1002/advs.202519574 (PMC13067868; doi:10.1002/advs.202519574)
Supplement: Supplementary file 1 — Supporting File: advs74209‐sup‐0001‐SuppMat.docx. [file ADVS-13-e19574-s001.docx]

**Supporting Information**

**
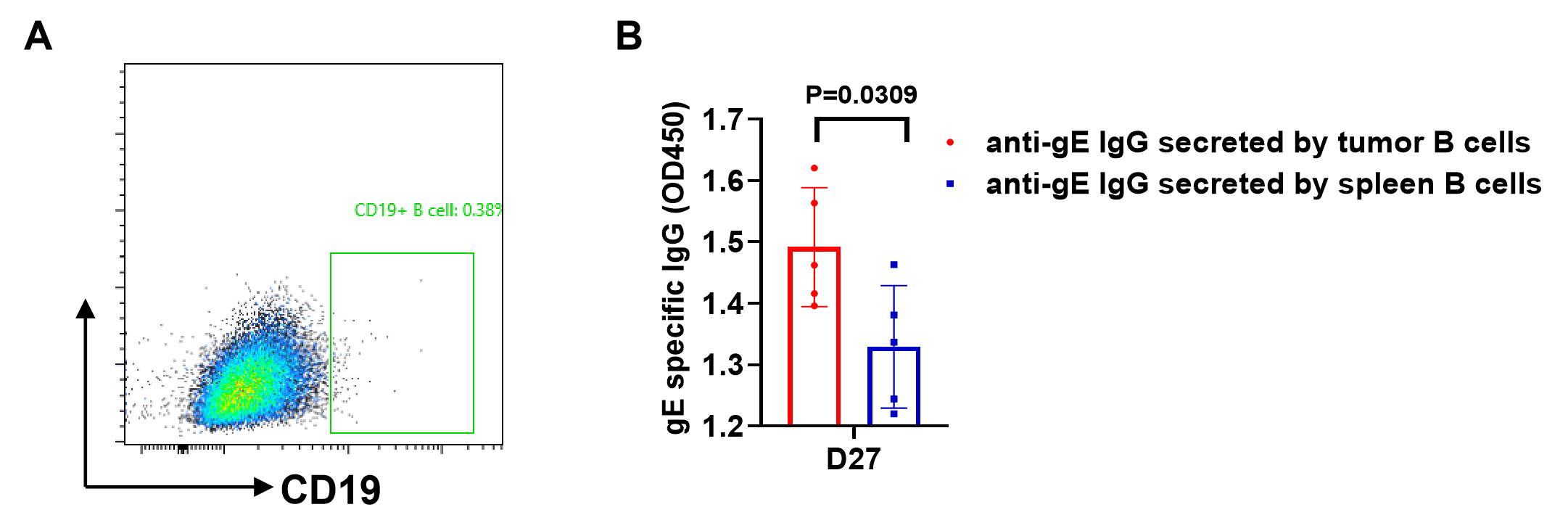
Figure S1. Enrichment of gE-specific B cells in tumor tissues of LZ901-immunized C57BL/6 mice.**

1. Flow cytometric analysis was performed to determine the proportion of B cells in the tumor tissues of C57BL/6 mice immunized with the recombinant zoster protein vaccine (LZ901).
2. CD19^+^ B cells were isolated from tumor tissue and spleen of LZ901-immunized C57BL/6 mice through magnetic bead separation. The isolated B cells were then stimulated with 5 ng/mL IL-4 and 2 μg/mL CD40L. The secretion of gE-specific IgG of B cells seperated from tumor and spleen was measured through ELISA. The results indicated that tumor B cells secret higher VZV glycoprotein E (gE) specific IgG than spleen B cells. Data are presented as the mean ± SD (n = 5). Statistical significance was determined using one-way ANOVA. Statistically significant differences were observed (P < 0.05).

**
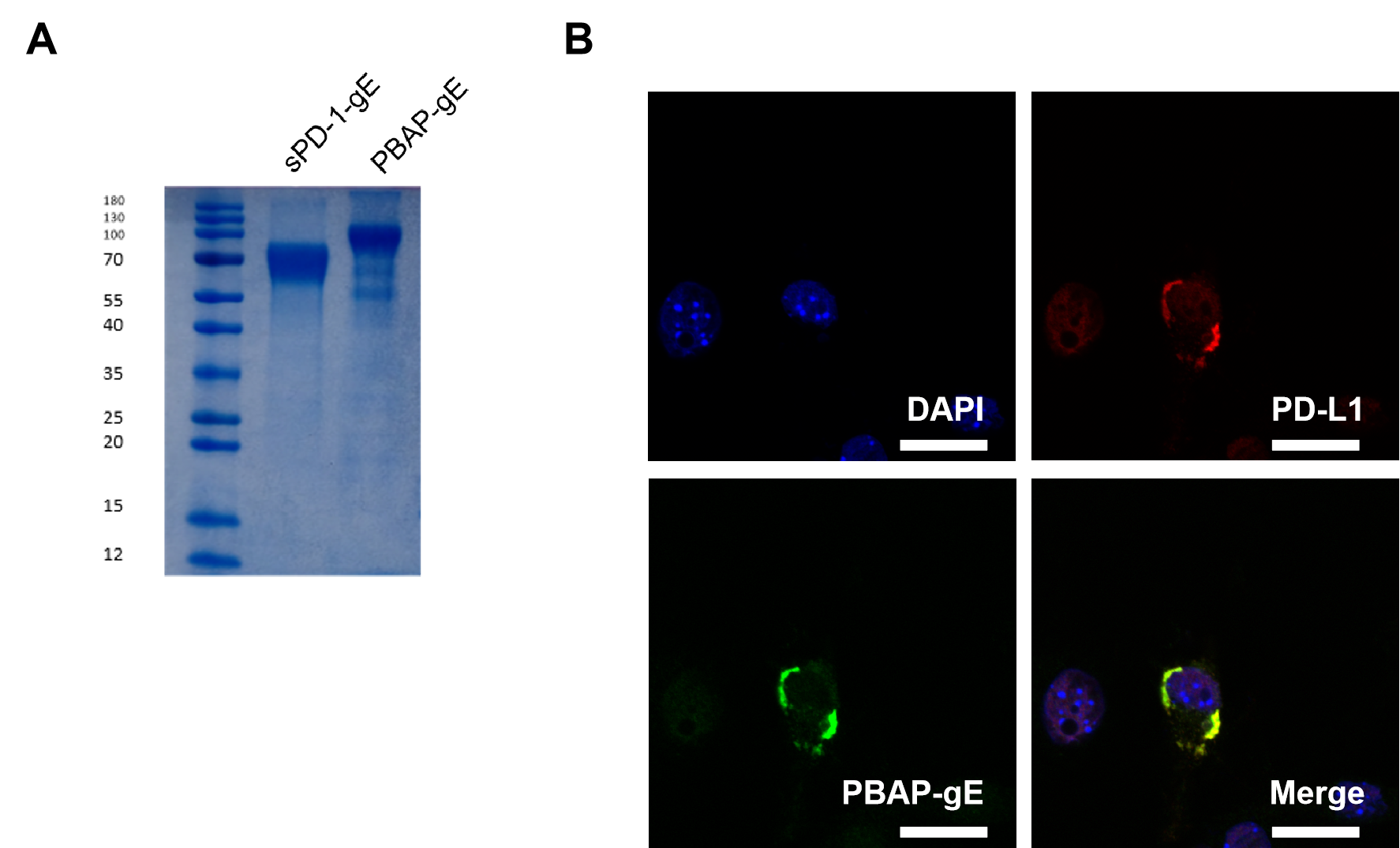
**

**Figure S2. Characterization of Recombinant Proteins sPD-1-gE and PBAP-gE.**

1. Coomassie Blue staining of sPD-1-gE and PBAP-gE verified the successful expression and high purity of the recombinant proteins.
2. Immunofluorescence staining reveals colocalization of PBAP-gE with endogenousc PD-L1 on 4T1 cells. Scale bars, 25 μm.

**Figure S3. PD-L1 Expression Analysis in 4T1 Cell Variants after IFN-γ Treatment and KIL C.2 Cell Cytotoxicity Against 4T1 Cell Variants**

1. Left panel: Cytotoxicity (expressed as percentage) of KIL.2 cells against 4T1-PD-L1-OE target cells, assessed across a gradient of effector-to-target (E:T) ratios (8:1 to 1:1). Cytotoxicity decreased with reducing E:T ratios, consistent with dose-dependent effector cell activity. Notably, the combination of KIL.2 cell with LZ901 vaccine serum + PBAP-gE maintained relatively higher cytotoxicity across E:T ratios compared to other groups. Right panel: Cytotoxicity of KIL.2 cells against 4T1-PD-L1-KO target cells. All data are presented as the mean ± SD (n = 3).
2. Flow cytometric analysis of PD-L1 surface expression in four 4T1-derived cell lines, assessed at 24 hours (left) and 48 hours (right) post-treatment with IFN-γ. Notably, PD-L1 expression was highest in 4T1-PD-L1-OE cells across both time points, while 4T1-PD-L1-KO cells showed minimal signal (consistent with the isotype control). IFN-γ treatment induced PD-L1 upregulation in 4T1-WT cells relative to the isotype control.

**
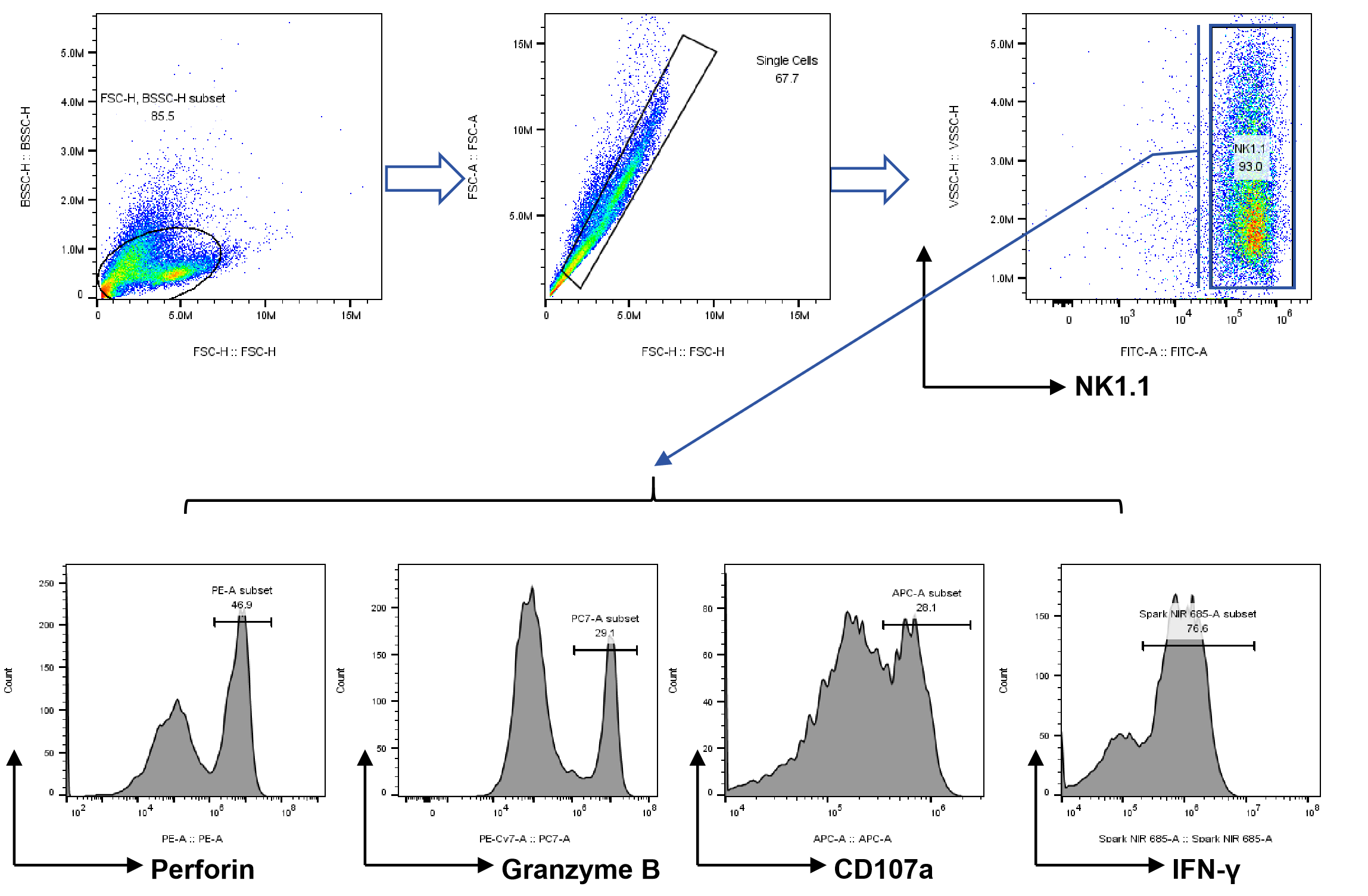
Figure S4. Flow Cytometric Gating Strategy and Activation Marker Analysis of Murine KIL C.2 Cells.**

Workflow for gating and functional marker assessment of murine KIL C.2 cells: debris is first excluded and single cells are selected via FSC/BSSC gating, followed by identification of the KIL C.2 cell population using the NK1.1⁺ phenotype. Finally, the expression of four functional markers (Perforin, Granzyme B, CD107a, and IFN-γ) is analyzed within this KIL C.2 subset. Data are representative of three independent experiments using samples from C57BL/6 mice.

**
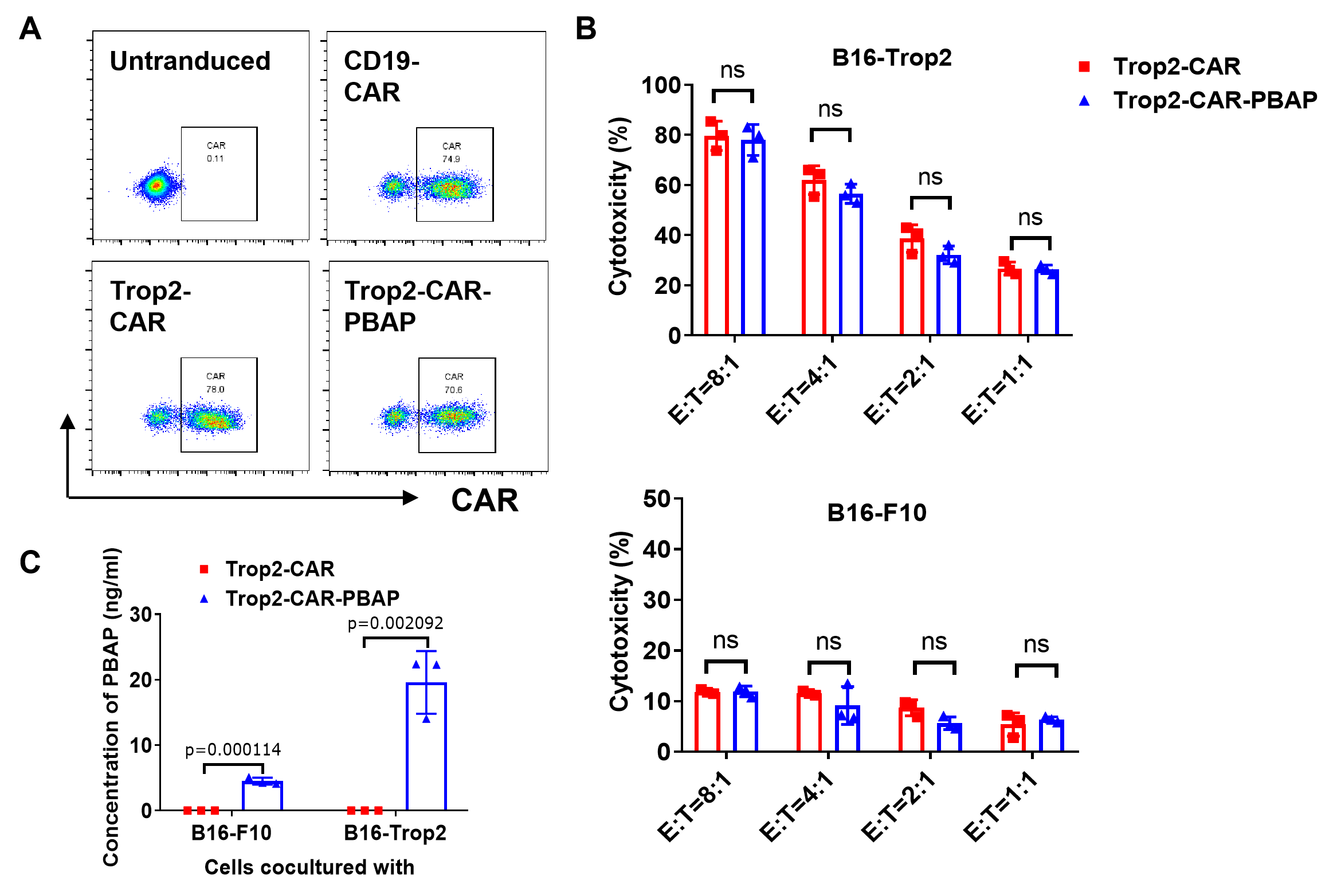
**

**Figure S5. Evaluation of Trop2-CAR and Trop2-CAR-PBAP Cells *in Vitro.***A) Flow cytometric analysis of chimeric antigen receptor (CAR) surface expression across four cell groups (including: untransduced group, CD19-CAR group，Trop2-CAR group, and Trop2-CAR-PBAP group).
B) Cytotoxicity assay. The cytotoxicity of Trop2-CAR and Trop2-CAR-PBAP cells against B16-Trop2 cells and B16-F10 cells was evaluated by LDH assay, B16-F10 cells were used as a negative control. Data are presented as the mean ± SD (n = 3). Statistical significance was determined using unpaired t test. ns indicates not significant (P > 0.05).

1. PBAP secretion analysis. The secretion of PBAP by Trop2-CAR-PBAP cells was measured upon co-culture with B16-F10 (negative control) and B16-Trop2 cells. Data are presented as the mean ± SD (n = 3). Statistical significance was determined using unpaired t test. ns indicates not significant (P > 0.05).

**
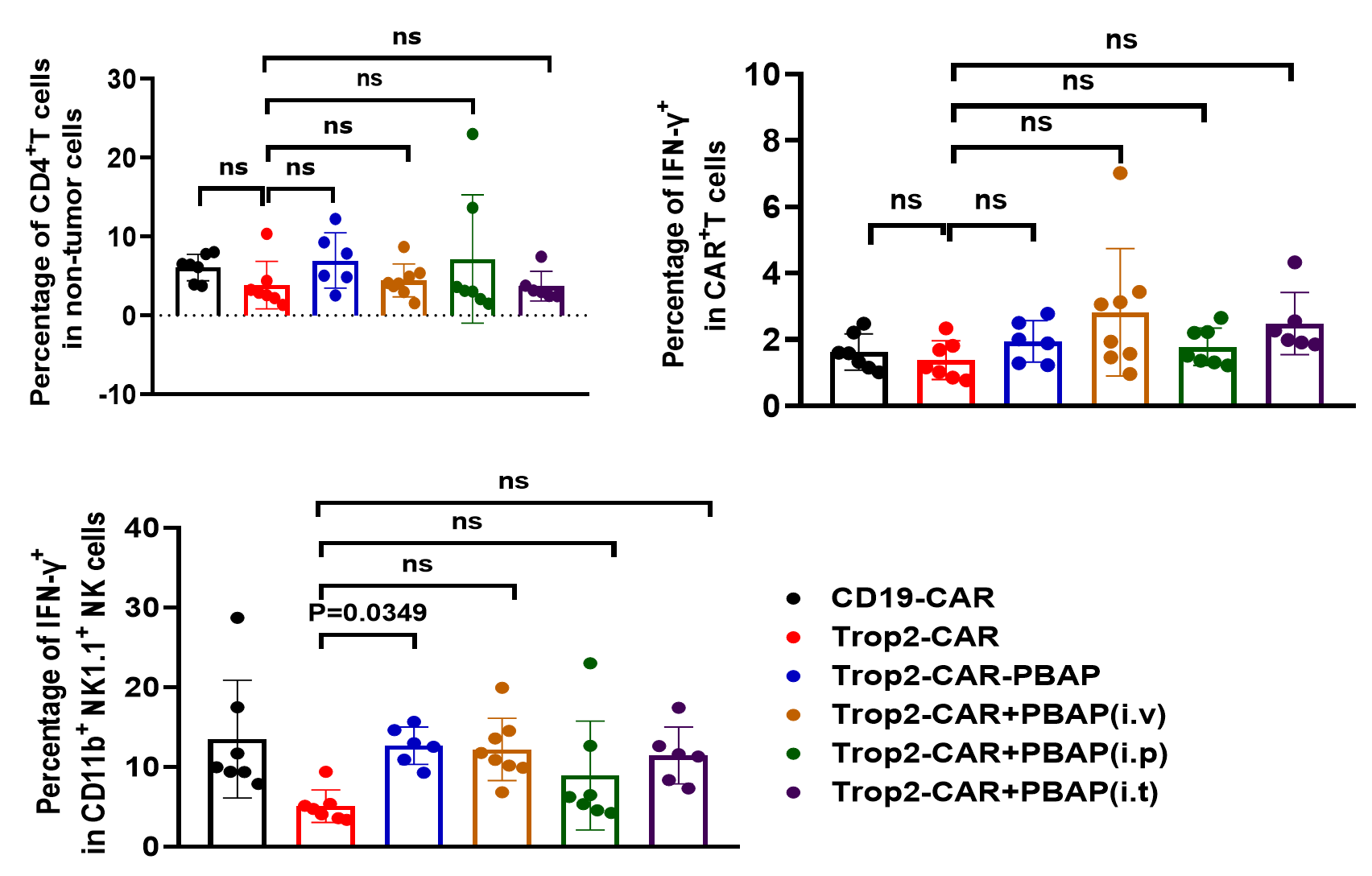
**

**Figure S6. Flow Cytometric Quantification of Tumor-Infiltrating Immune Cells.**

Quantification of three immune cell populations across six treatment groups (CD19-CAR, Trop2-CAR, and Trop2-CAR-PBAP via *i.v./i.p./i.t.* routes): (1) CD4^+^ T cell percentage in non-tumor cells (ns between groups); (2) IFN-γ^+^ subset proportion in CAR-T cells (ns between groups); (3) IFN-γ^+^ subset proportion in CD11b^+^ NK1.1^+^ NK cells (only CD19-CAR vs. Trop2-CAR showed a significant difference, P = 0.0349; other comparisons were ns). Data are presented as the mean ± SD (n =6-8). Statistical significance was determined using one-way ANOVA. Each dot denotes an individual sample, error bars reflect group variability, and ns indicates not significant (P > 0.05).

**
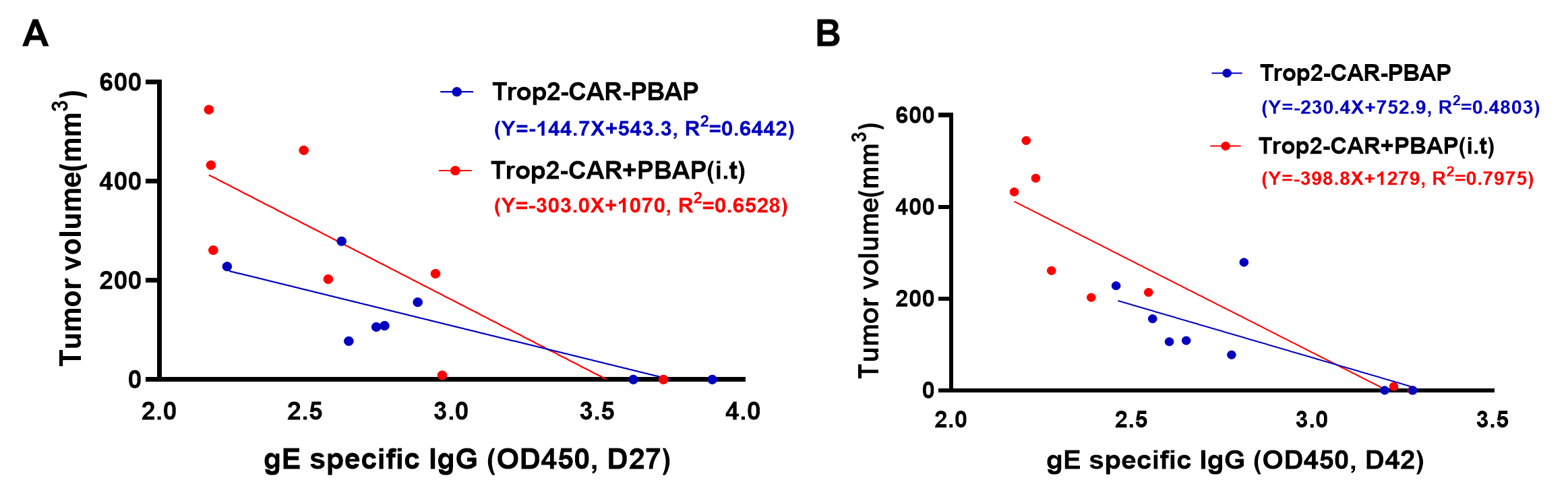
**

**Figure S7. The Tumor Volume is Negatively Correlated with gE-specific IgG Antibody Levels.**

Correlation analysis of tumor volume with gE-specific IgG antibody levels (OD450) at days 27 and 42 between Trop2-CAR-PBAP treatment group and Trop2-CAR + PBAP treatment group.

**Figure S8. PBAP-gE Elicits a Robust Anti-Tumor Immune Response, Predominantly Mediated by NK Cells through gE-Specific Antibody Production by B Cells.**

1. Experimental design to assess the contributions of antibody versus CD8^+^ T cells in PBAP-gE-mediated tumor suppression. C57BL/6J mice (n =3-5 mice/group) were immunized with the LZ901 vaccine (5 μg/dose) on days 0 and 21. On day 28, 5 × 10^5^ B16-fLuc cells (luciferase-IRES-RFP⁺) were subcutaneously engrafted. B cell depletion was achieved on day 33 via anti-CD19, anti-CD22, and anti-B220 antibodies, while CD8^+^ T cell depletion was induced by anti-CD8 antibodies. On day 35, mice received intratumoral injections of PBAP-gE (150 μg/mouse), and tumor growth was monitored using an *in vivo* imaging system (IVIS), allowing for real-time tracking of tumor dynamics. Created with BioRender.com.
2. In tumor-bearing mice, *in vivo* imaging results before and after depletion of B cells or CD8^+^ T cells were obtained. The group with depletion of B cells and the group with depletion of CD8^+^ T cells each included 5 mice, while the control group contained 3 mice. Quantitative analysis of total tumor flux (normalized to day 34) over time. The B cell Block group showed significantly increased tumor growth compared to the control group at day 40 (P=0.0113), while the CD8^+^ T cell block group did not exhibit a significant difference. Error bars represent variability within groups. Data are presented as the mean ± SD. Statistical significance was determined using Kruskal-Wallis test. ns indicates not significant (P > 0.05).

**
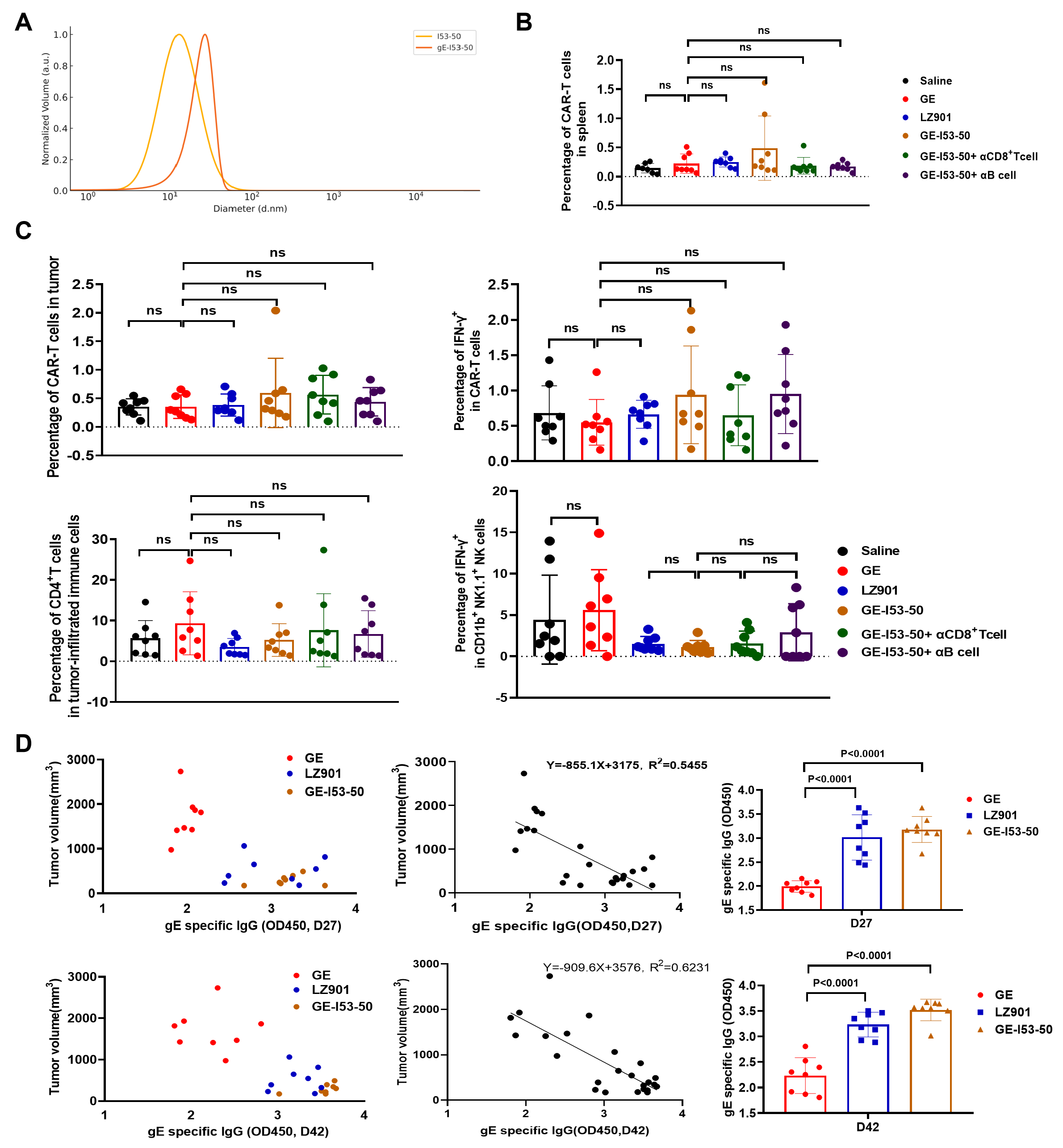
Figure S9. Nanoparticle Characterization, Immune Cell Profiling, and Correlation Analysis of Tumor volume with gE specific IgG.**

A) Particle size distribution analysis of GE-I53-50 and I53-50 nanoparticles.

B) Flow cytometric analysis of percentage of CAR-T cells in splenocytes across six groups (Saline, GE, LZ901, GE-153-50, GE-153-50+αCD8⁺ T cell, GE-153-50+αB cell); no significant differences (ns) were observed between groups. Data are presented as the mean ± SD (n = 8). Statistical significance was determined using one-way ANOVA. ns indicates not significant (P > 0.05).

C) Flow cytometric analysis of CAR-T cells in tumor, IFN-γ^+^ proportion in CAR-T cells, CD4^+^ T cells in tumor-infiltrating immune T cells and IFN-γ^+^ cells in CD11b^+^NK1.1^+^ NK cells. Data are presented as the mean ± SD (n = 8). Statistical significance was determined using one-way ANOVA. ns indicates not significant (P > 0.05).

D) Correlation analysis of tumor volume with gE-specific IgG antibody levels (OD450) at days 27 and 42 among the GE group, LZ901 group, and GE-I53-50 immunization group. Data are presented as the mean ± SD (n = 8). Statistical significance was determined using one-way ANOVA. ns indicates not significant (P > 0.05).

**
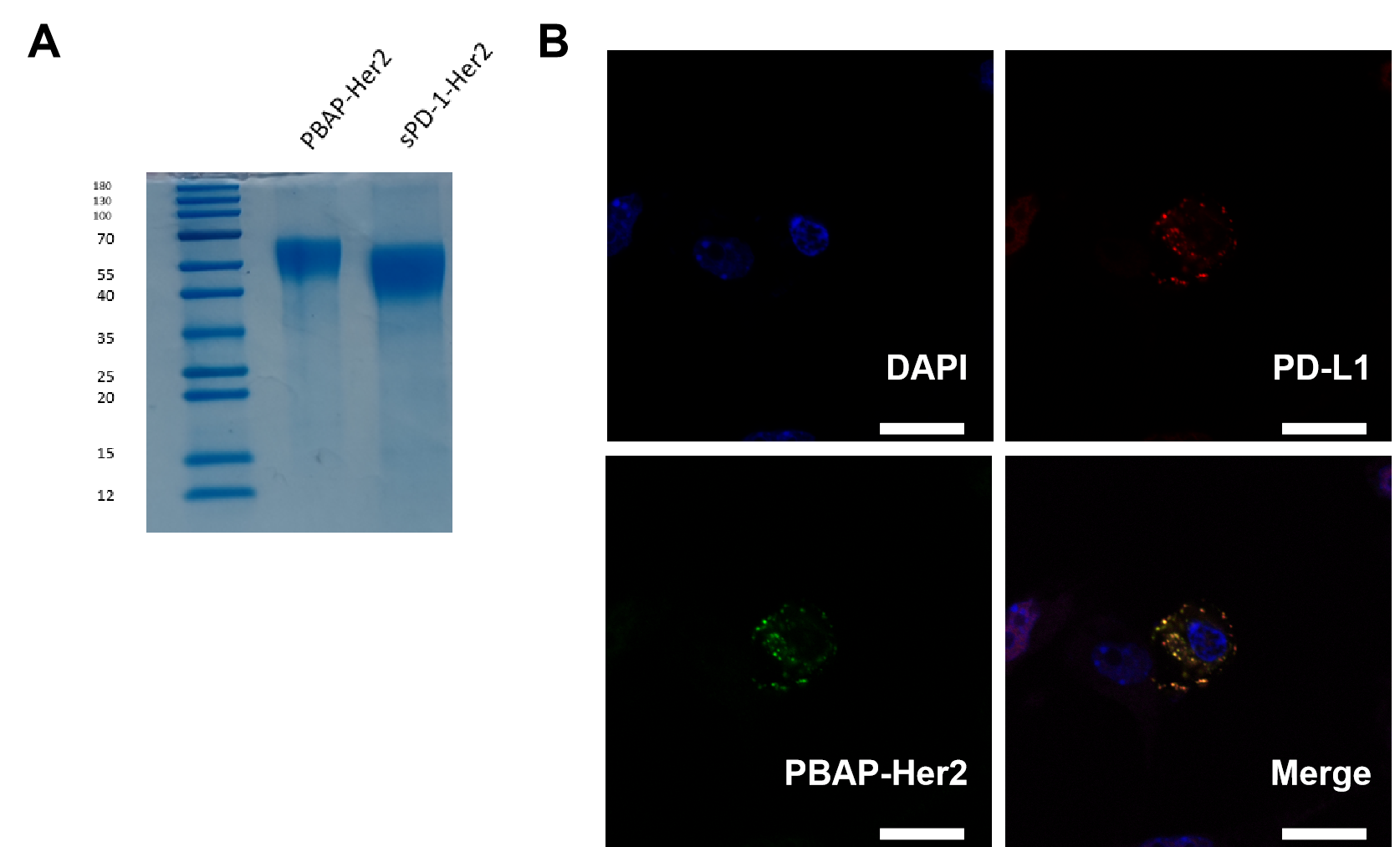
**

**Figure S10. Characterization of Recombinant Proteins PBAP-Her2 and sPD-1-Her2.**

1. Coomassie Blue staining of sPD-1-Her2 and PBAP-Her2 verified the successful expression and high purity of the recombinant proteins.
2. Immunofluorescence staining reveals colocalization of PBAP-Her2 with endogenousc PD-L1 on MDA-MB-231 cells. Scale bars, 25 μm.

**Figure S11. PD-L1 Expression in MDA-MB-231 Cell Variants after IFN-γ Treatment and Cytotoxicity of NK Cell Against MDA-MB-231 Cell Variants.**

1. Flow cytometric analysis of PD-L1 surface expression in four MDA-MB-231-derived cell lines, assessed at 24 hours (left) and 48 hours (right) post-treatment with IFN-γ. Notably, PD-L1 expression was highest in MDA-MB-231-PD-L1-OE cells across both time points, while MDA-MB-231-PD-L1-KO cells showed minimal signal (consistent with the isotype control). IFN-γ treatment induced PD-L1 upregulation in MDA-MB-231-WT cells relative to the isotype control.
2. Left panel: Cytotoxicity (expressed as percentage) of NK cells against MDA-MB-231-PD-L1-OE target cells, assessed across a gradient of effector-to-target (E:T) ratios (8:1 to 1:1). Cytotoxicity decreased with reducing E:T ratios, consistent with dose-dependent effector cell activity. Notably, the combination of NK cell with Trastuzumab + PBAP-HER2 maintained relatively higher cytotoxicity across E:T ratios compared to other groups. Right panel: Cytotoxicity of NK effector cells against MDA-MB-231-PD-L1-KO target cells. All data are presented as the mean ± SD (n = 3).

**
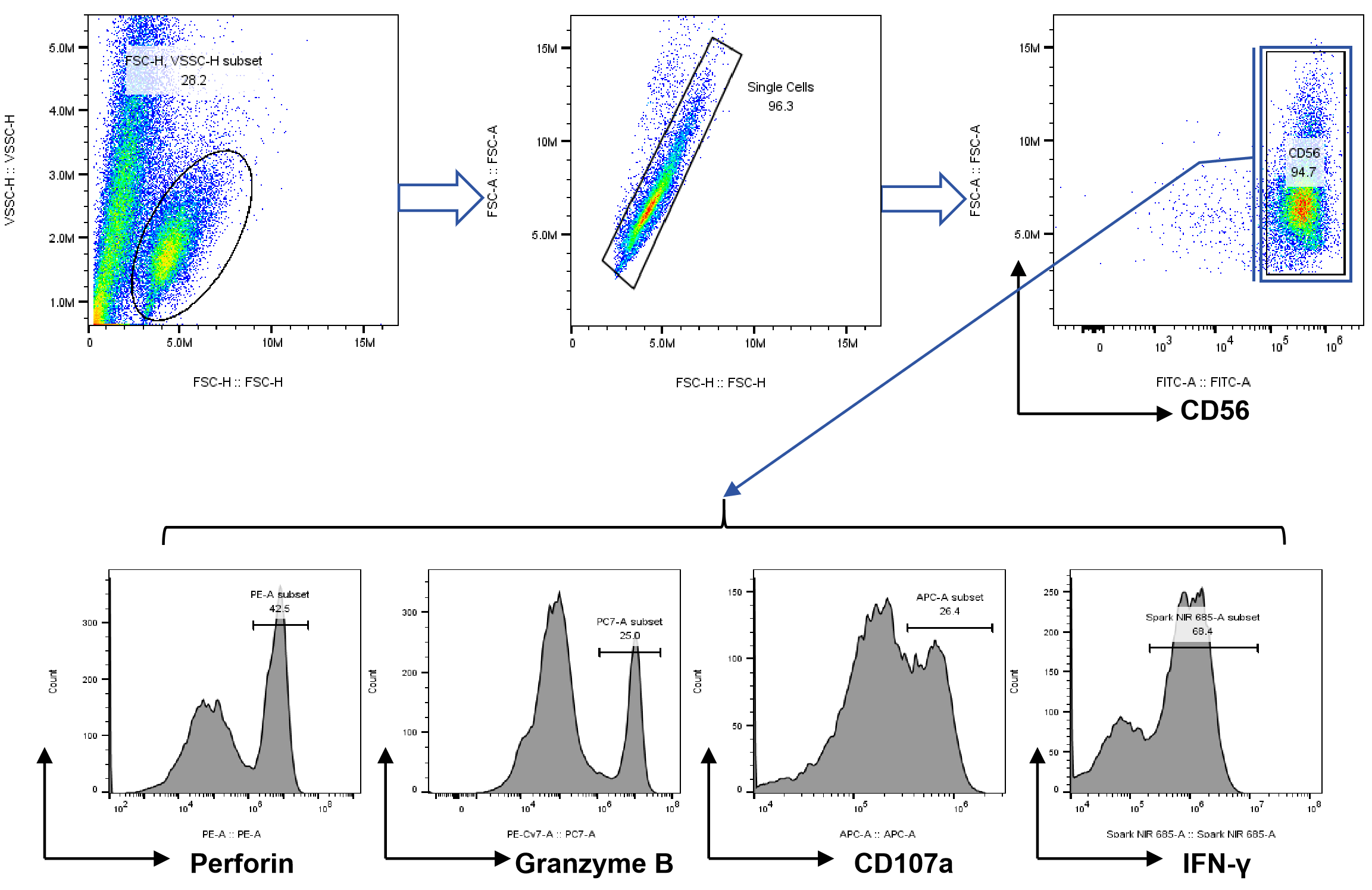
Figure S12. Flow Cytometric Gating Strategy and Activation Marker Analysis of Human Peripheral Blood NK Cells.**

Workflow for gating and activation assessment of human peripheral blood NK cells: debris is first excluded and single cells are selected via FSC/VSSC gating, followed by identification of the NK cell population using the CD3⁻CD56⁺ phenotype. Finally, the expression of four activation-associated markers (Perforin, Granzyme B, CD107a, and IFN-γ) is analyzed within this NK cell subset. Data are representative of three independent experiments using peripheral blood samples from healthy donors.

**
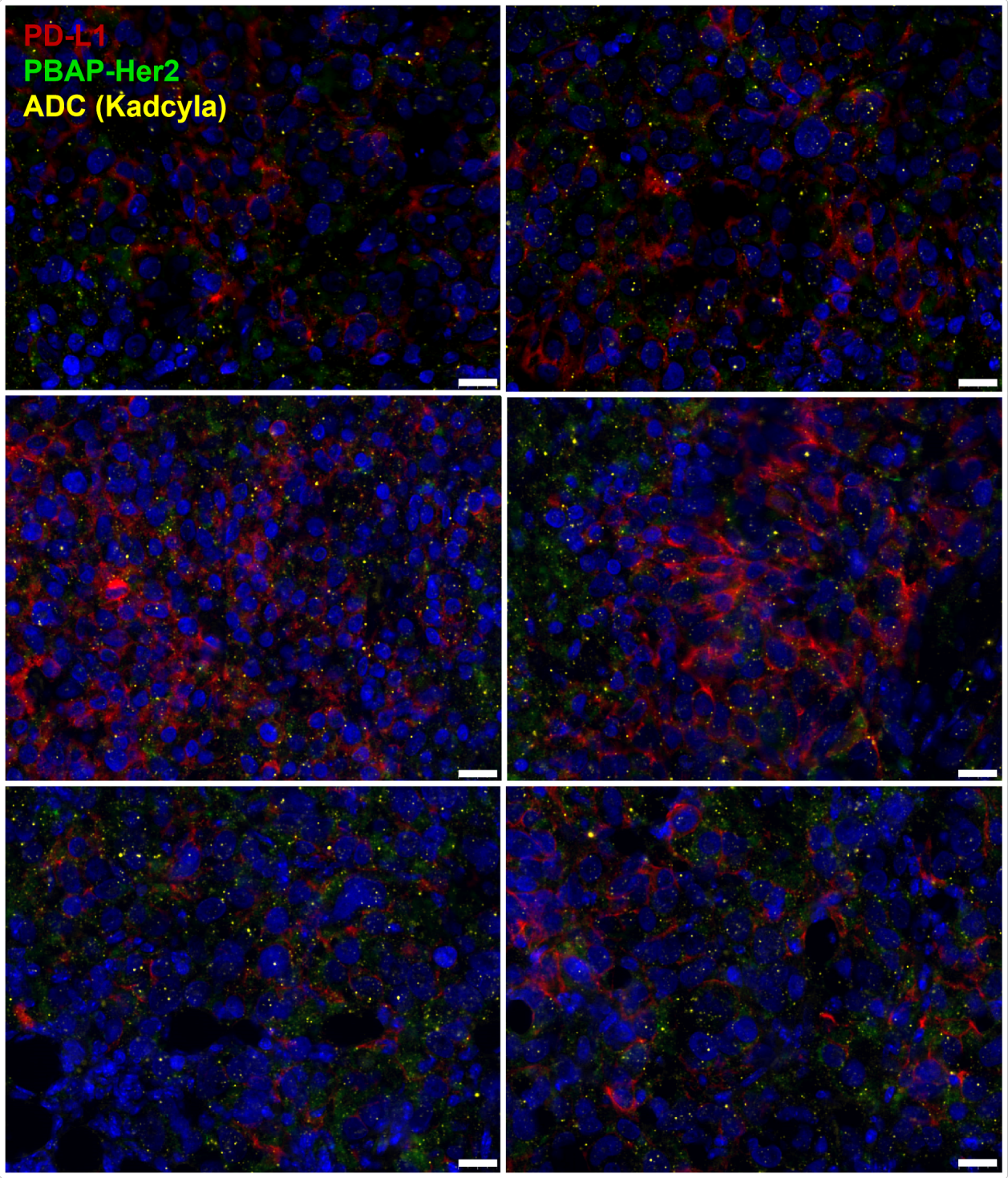
Figure S13. PBAP-Her2 Synergizes with Antibody-Drug Conjugate Kadcyla in NSG Mice Bearing Subcutaneous Tumors.**

Representative immunostaining images for PD-L1, PBAP-Her2, and Antibody-Drug Conjugates (ADC) Kadcyla. Nuclei are shown in blue with DAPI. Scale bars, 20 μm.
